# A Multi-Pathway Approach to Pesticide Exposure in Agricultural Communities: Protocol for an Observational Study

**DOI:** 10.2196/94740

**Published:** 2026-08-21

**Authors:** Raphaëlle Teysseire, Jonathan Chevrier, Pedro Alejandro Segura, Benoit Barbeau, Amadou Barry, Jacques Brodeur, Louise Hénault-Ethier, Marc-André Verner, Maryse F Bouchard

**Affiliations:** 1 Unité d'épidémiologie et de biostatistique Centre Armand-Frappier Santé Biotechnologie Institut National de la Recherche Scientifique Laval, QC Canada; 2 Department of Epidemiology, Biostatistics and Occupational Health Faculty of Medicine and Health Science McGill University Montréal, QC Canada; 3 Département de chimie Université de Sherbrooke Sherbrooke, QC Canada; 4 Department of Civil, Geological and Mining Engineering Polytechnique Montréal Montréal, QC Canada; 5 Unité mixte de recherche INRS-UQAC en santé durable Institut National de la Recherche Scientifique Chicoutimi, QC Canada; 6 Department of Occupational and Environmental Health School of Public Health Université de Montréal Montréal, QC Canada; 7 Centre Eau Terre Environnement Institut National de la Recherche Scientifique Québec, QC Canada

**Keywords:** pesticide, resident, exposure assessment, biomonitoring, environmental monitoring

## Abstract

**Background:**

The widespread use of pesticides in agriculture raises concerns about exposure among populations living near treated fields. Environmental contamination may lead to prolonged pesticide exposure, with potential health effects, yet significant knowledge gaps remain regarding exposure levels and pathways in agricultural communities.

**Objective:**

The Exposure to Pesticides Used in Regions of Agriculture (EPURA) study aims to characterize pesticide exposure patterns and identify their key sources and determinants through an integrated approach combining biological and environmental measurements.

**Methods:**

We recruited 400 participants from agricultural communities living within 1500 meters of vegetable production fields in Quebec, Canada, throughout the 2 years of data collection. Over 4 summer months and 3 winter months, participants provided monthly urine samples and floor wipes and completed questionnaires on diet, water consumption, household characteristics, pesticide use, and lifestyles. Summer and winter urine samples were pooled for each participant, and summer and winter wipe samples were pooled for each household. At the end of the summer, we collected additional samples, including indoor dust, yard soil, and tap water. Environmental and biological samples will be analyzed for 51 pesticides and their metabolites. Pesticide applications near residences will be estimated using agricultural land use data integrated into a geographic information system. Pesticide intake from food and water will be assessed by combining consumption data with pesticide residues measured in water and in food monitoring programs. Residential contamination will be estimated by measuring pesticides in indoor dust and yard soil. Urinary pesticide concentrations will be used to estimate exposure doses. Urinary concentrations will be compared across population subgroups and seasons and with available Canadian biomonitoring data. Associations between exposure doses and dietary and water intake, indoor and outdoor contamination, proximity to treated fields, and household-level determinants will be examined using mixed models. Variable selection will be performed using high-dimensional methods.

**Results:**

As of June 1, 2026, participant recruitment and data collection are complete. A total of 173 households (372 participants) were included in the study, with 1049 wipe samples, 170 sealed petri dishes, 157 water samples, 170 yard soil samples, and 2257 urine samples collected. Completion of all laboratory analyses is expected in 2027, after which the statistical analyses will be conducted in accordance with the study objectives.

**Conclusions:**

This study will generate crucial data on pesticide exposure in agricultural communities. Its findings will help identify major determinants of household contamination and key risk factors contributing to residents’ pesticide exposure. Ultimately, the EPURA study will support the development of targeted strategies to reduce pesticide-related health risks and inform public health policies.

**International Registered Report Identifier (IRRID):**

DERR1-10.2196/94740

## Introduction

According to the Food and Agriculture Organization, global pesticide use in agriculture reached 3.7 million tons of active ingredients in 2022, doubling since 1990 [1]. Intensive conventional agriculture raises serious concerns about its environmental and health impacts, particularly for communities living near fields where pesticides are heavily applied. These populations face long-term environmental exposure to complex mixtures of chemical substances. Epidemiological studies have linked proximity to treated fields with an increased risk of some cancers, neurological disorders, adverse pregnancy outcomes, developmental delays in children, and respiratory issues [2,3].

During and after pesticide application on crops, a significant portion of these chemicals can disperse into the surrounding environment through air, soil, or water, potentially reaching nearby residential areas and thereby exposing the rural population. Recent studies have found associations between proximity to agricultural fields, crop density, and local pesticide use and elevated pesticide concentrations, including organochlorines, organophosphates, or pyrethroids, in indoor dust and residents’ urine. These studies also indicate that some pesticides’ concentrations tend to peak during high-application seasons [4-9].

Although research on pesticide exposure in agricultural communities has increased in recent years, substantial data gaps persist [10]. Exposure occurs through multiple pathways, including inhalation; dermal contact; and ingestion of contaminated food, water, soil, or dust (the latter being particularly relevant for children through frequent hand-to-mouth and object-to-mouth behaviors). However, most studies have focused on specific media, mostly urine and indoor dust, limiting the capacity to identify all exposure pathways and their relative contributions. Moreover, the determinants of residential contamination and individuals’ exposure to pesticides remain poorly understood. In addition to seasonal variations in pesticide use, other influencing factors may include landscape characteristics, weather conditions, housing features, and occupants’ lifestyles. Exposure levels, sources, and determinants may also vary significantly by geographic location given differences in climate, meteorology, crop types, pesticide regulations, and population characteristics. Most research has been conducted in the United States and some European countries, and their findings may not be directly applicable to other regions. Understanding exposure levels, sources, and determinants across different agricultural communities is essential for developing targeted prevention strategies.

In Canada, fungicide, herbicide, and insecticide use has increased rapidly and systemically over the past decades, particularly in the Prairies and the central regions, including Quebec [11,12]. However, data on pesticide exposure and health impacts in agricultural communities are scarce. A study that estimated potential pesticide exposure throughout Canada reported that Quebec was the province with the largest number of highly exposed individuals for glyphosate-containing herbicides and for 2,4-dichlorophenoxyacetic acid [13]. These findings highlight the need for direct measurement of pesticide exposure in Quebec to address existing knowledge gaps.

The Exposure to Pesticides Used in Regions of Agriculture (EPURA) study (2023-2028) aims to quantify pesticide exposure in individuals living near vegetable-growing fields by collecting multiple biological and environmental samples. Specifically, the study will measure pesticide residues in participants’ urine, assess pesticide levels both inside and outside their homes, evaluate pesticide concentrations in drinking water, estimate pesticide intake through diet, and examine the influence of the proximity and intensity of local agricultural activities on pesticide exposure. The EPURA study focuses on vegetable production because of its intensive pesticide use and the wide variety of chemicals applied, typically present as mixtures. Additionally, toxic pesticides for humans, such as insecticides and fungicides, are commonly used in this sector [14].

## Methods

### Study Design

#### Study Sites

The study is being conducted in Montérégie, a region in southern Quebec characterized by intensive agricultural activity and rapidly expanding residential development. The agricultural area spanned 953,402 hectares in 2014, representing approximately 86% of the region’s territory, with nearly 60% of this land under cultivation. Montérégie is the largest agricultural region in Quebec, leading the province in the production of fruits and vegetables as well as cereals and oilseeds [15]. Pesticides and their transformation products have been detected in both drinking and surface waters in the region, particularly during the pesticide spraying period [16].

Study sites targeted for inclusion in the study were towns with a high density of vegetable crops in close proximity to residential areas. To identify these towns, we used 2023 and 2024 agricultural land use data from the Parcels and Reported Agricultural Productions Database of La Financière agricole du Québec, an organization that provides income protection, insurance, and financing services to the agricultural sector [17]. Using QGIS (QGIS Development Team), an open-source geographic information system [18], we created 1500-m buffers around mapped vegetable crop parcels and selected 4 towns with a high concentration of residences near these crops, identified through the Quebec building reference open data file (Référentiel québécois sur les bâtiments) edited by the Quebec government [19]. Information on the number of inhabitants and residences in the eligible municipalities was obtained from the 2021 Canadian census data [20].

#### A Community-Inclusive Approach

The EPURA study has adopted a community-inclusive approach by establishing an advisory committee that is involved in all phases of the research, including refining the research questions, developing protocols and questionnaires, and interpreting and communicating the results. The advisory committee includes 8 community members representing a range of age groups and literacy levels actively involved in community life. Its membership reflects diverse local stakeholders, including municipal advisors, farmers, health professionals, and citizens.

#### Population Recruitment

We aimed to recruit 400 individuals living within 1500 meters of vegetable fields. All full-time residents of the selected municipalities were eligible regardless of age or occupation, and multiple household members may be enrolled. Recruitment was conducted in 2024 and 2025 through mailed flyers, posters displayed in towns, social media, municipal newsletters, and community events. This approach maximized the diversity of recruited participant profiles and further reduced the risk of systematic selection toward any particular subgroup of the population.

A participation rate of approximately 5% was anticipated in the identified sites based on participation rates observed in 2 previous studies involving residents near agricultural fields [21,22]. Recruitment was launched in 2024 in 2 towns as a planned first phase, serving both to estimate the local participation rate and inform any necessary adjustment to the number of study sites in 2025, an adaptive approach designed to ensure that the target sample size would be met.

### Overall Approach

#### Biomonitoring

Exposure of individuals to pesticides will be assessed for summer and winter through measurements in urine samples. Biomonitoring of pesticide residues and their urinary metabolites captures exposure from multiple routes, including ingestion, inhalation, and dermal absorption. Multiple urine samples were collected from each participant in each season to provide a more reliable assessment of long-term exposure despite the short biological half-life of most targeted pesticides in this study [23].

#### Assessment of Nondietary Sources

Nondietary sources of exposure will be evaluated through environmental measurements. Indoor contamination will be assessed by measuring pesticides in 2 types of samples. First, dust deposits were collected throughout the summer in petri dishes [24]. In addition, solvent-wetted wipes were used by participants to collect pesticide residues from the floor at the entrance of the house multiple times during both summer and winter [22,25]. As floors are regularly cleaned, this sampling was expected to reflect recent contamination, unlike petri dishes, which capture contamination over several months. Outdoor contamination will be evaluated by measuring pesticide concentrations in soil samples collected in the yard for participants who had access to one.

#### Assessment of Dietary Sources

In Quebec, pesticide monitoring in municipal drinking water systems is limited to a few pesticides at specific water supply sites. Moreover, many residences in rural areas rely on private wells not equipped for pesticide removal and not subject to monitoring. In our study, pesticide intake from drinking water will be estimated by measuring pesticides in tap water and linking these results to participants’ reported water consumption.

We will estimate dietary pesticide intake using participants’ food consumption data collected using a questionnaire and pesticide contamination information from the National Chemical Residue Monitoring Program of the Canadian Food Inspection Agency [26] and the annual pesticide residue monitoring results for fresh fruits and vegetables of the Quebec Ministry of Agriculture, Fisheries, and Food [27]. This approach has been used to estimate pesticide ingestion from diet in previous studies [28,29].

#### Potential Factors Influencing Exposure and Contamination

We collected information on factors related to the residence and its occupants that may influence pesticide concentrations in biological and environmental samples, including the home’s layout, household characteristics, and the residents’ lifestyles.

Currently, spatialized data on pesticide use in Quebec are unavailable. We will calculate surrogate indicators of the spatial intensity of agricultural pesticide use, such as the residential distance to the nearest treated field or the area of treated fields near the home, for each pesticide quantified in the samples. These indicators will be calculated using QGIS based on land use data from the Parcels and Reported Agricultural Productions Database or the annual Agriculture and Agri-Food Canada annual crop inventory that provides satellite-based mapping of crops [30]. If available, we will incorporate into the development of these indicators qualitative or quantitative data on pesticide use collected from experts within the Quebec Plant Protection Warning Network [31] or local agricultural advisors.

The effect of seasons will also be investigated, with summer representing the peak period of pesticide application, whereas no pesticide use is expected during the winter in Quebec due to climatic constraints (low temperatures, snow cover, and frozen soil).

**Figure 1:**
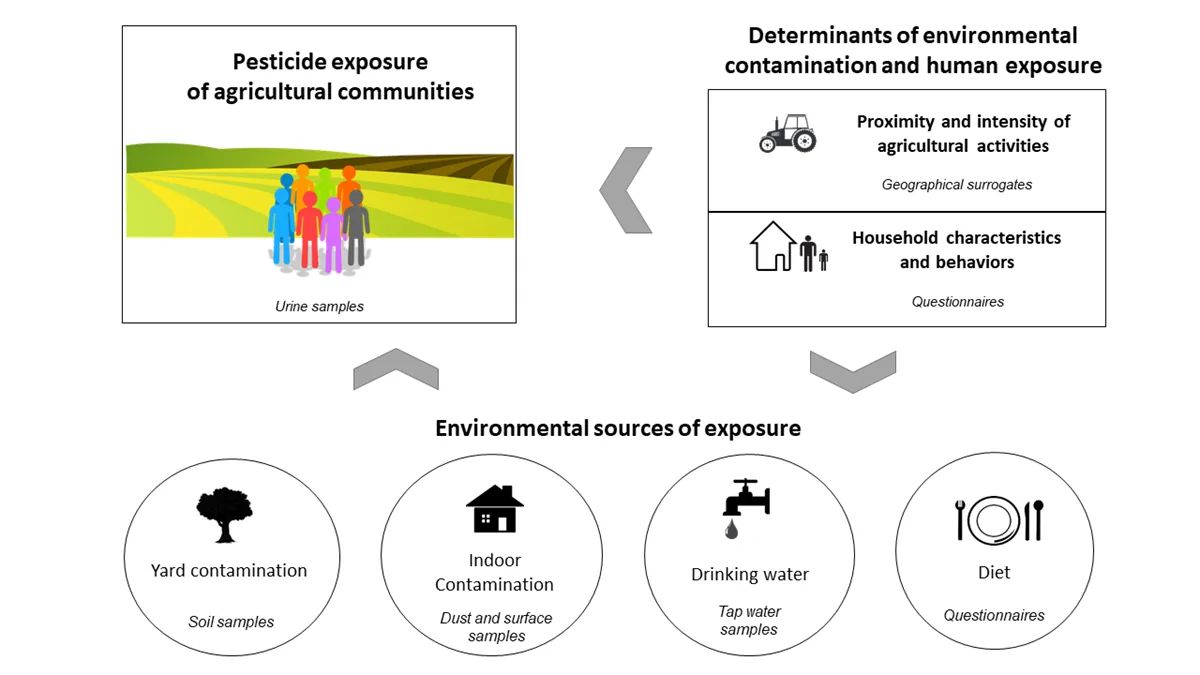
Integrated approach to analyzing levels, sources, and determinants of pesticide exposure in agricultural communities.

Figure 1 summarizes the overall approach of the EPURA study to assess pesticide exposure levels affecting agricultural communities in Quebec and identify its main sources and determinants.

### Sample Collection

#### Urine

During an initial home visit, participants were given 120-mL urine collection containers and instructed to collect the first-morning urine sample once a month in summer (May to August) and again once a month in winter (January to March). All members of the household were asked to collect their urine on the same day. The urine samples were stored in the household’s freezer until they were retrieved by the research team and then transported in a cooler with dry ice to the laboratory for storage at −80 °C on the same day. Before analysis, a pooled summer urine sample and a pooled winter urine sample will be prepared for each participant.

#### Floor Surface Samples

One of the household members wiped the entryway floor of the home 4 times in the summer and 3 times in the winter on the same days as the urine collection. During the initial visit, a research assistant instructed the participants on the sampling locations, giving priority to the floor near the main entrance within 2 meters of the door. Samples were collected using a sampling kit provided during the visit after the participant had first washed their hands with soap. The kit included premoistened (91% isopropyl alcohol and 9% deionized water) wipes (CleanTex TexPad tape head wipes), a 900-cm^2^ cardboard template, and aluminum bags to store the wipes after sampling. A demonstration of how to wipe the floor (making 3 S-shaped wipes and folding the cloth between each) was given by the research assistant. Each wipe was placed in an aluminum bag and stored in the participant’s freezer until it was retrieved and transported by the research team in a cooler with dry ice for storage at −20 °C on the same day. Regarding urine, 1 summer and 1 composite sample will be created for each household during extraction.

#### Indoor Dust

Dust from each home was collected over several months using an open 15-cm-diameter glass dish and its lid. The dish and lid were placed at a height of approximately 2 meters on a piece of furniture in the main living area during the initial home visit (May) and retrieved at the end of the summer (August to September). Once closed, the petri dish was sealed with parafilm or adhesive tape and placed on dry ice in a cooler for transport to the laboratory, where it was stored at −20 °C.

#### Soil From the Yard

Soil was collected at the end of the summer from 5 sites in the yard of the residence. The sampling covered different zones of the property, including areas frequently used by household members (eating areas, vegetable gardens, and children’s play areas). Five volumetrically equivalent samples of the topsoil (0-5 cm) were collected using an auger after removing the grass layer with a flat spade. The collected soil was placed directly into a prelabeled polyethylene bag and then homogenized to create a composite sample. The sample was immediately placed in a cooler with dry ice for transport to the laboratory for storage at −20 °C.

#### Tap Water

Tap water (800 mL) from each participant’s kitchen was collected by a research team member at the end of the summer using a 1-L high-density polyethylene bottle. The sampling bottle was rinsed 3 times with tap water before collection. If the residence had 2 separate taps for drinking water and water used for food preparation with different water treatment methods, samples were collected from both taps.

### Questionnaires

#### Self-Administered Questionnaires

On each day of urine and floor sample collection, participants completed questionnaires that collected information about time spent in various locations (eg, home, yard, school, and other outdoor locations within the municipality), activities performed during that day and the previous week, water consumption, the origin and preparation methods of consumed food items, and pesticide use at home or work. For agricultural workers, the questionnaire also included questions about farming activities carried out the previous week and whether participants wore work shoes or clothing inside the home. The self-administered questionnaires are provided in Multimedia Appendix 1.

#### Self-Administered 24-Hour Diet Recalls

On each day of urine and floor sample collection, participants also completed a self-administered 24-hour diet recall online. The Automated Self-Administered 24-hour Dietary Assessment Tool was used [32]. The Automated Self-Administered 24-hour Dietary Assessment Tool–Canada-2018 version has been adapted and validated for the Canadian population and is available in French, the primary language spoken by the participants.

#### Household Questionnaire

At the end of the summer, a structured questionnaire was administered by a research team member to 1 household member on the same day as soil, dust, and water sample collection*.* The questionnaire gathered information on the household’s sociodemographic characteristics (participants’ age, sex, educational level, profession, and income), details about the residence (type, size, ventilation system, and water source and treatment), hygiene practices (cleaning methods and frequency, airing habits, shoe removal, presence of doormats, pets, and laundry drying in the yard), activities in the yard during the season, and the use of domestic pesticides both inside and outside the home. The household questionnaire is available in Multimedia Appendix 2.

**Figure 2:**
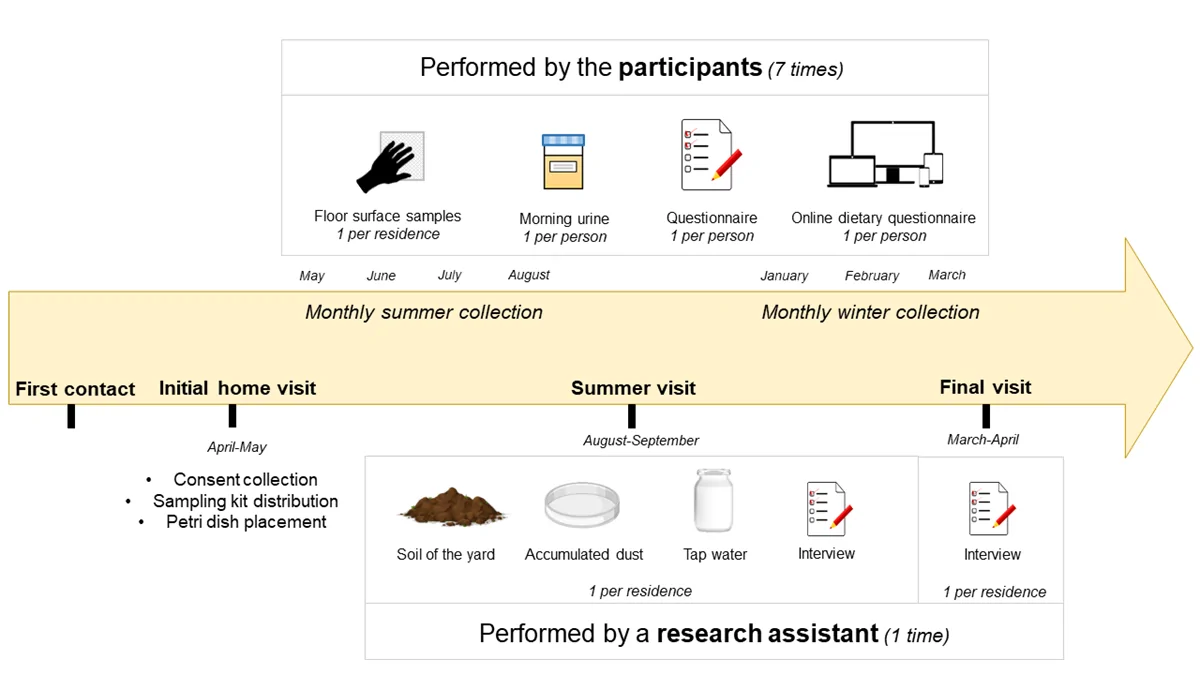
Strategies for survey administration and urine and environmental sampling.

Figure 2 shows the pesticide sampling strategy used in the EPURA study, including the collection of urine and environmental samples from participants, as well as the survey conducted to gather data from the study population.

### Pesticide Analysis

#### Pesticide Selection

The selection criteria for pesticides analyzed in the EPURA study were registration status in 2023 (being approved), intensity of local use based on experts, toxicity to the environment or humans, analytical feasibility, and budget constraints of the project.

A total of 51 parent compounds, consisting of 18 (35.3%) fungicides, 19 (37.3%) herbicides, and 14 (27.5%) insecticides, will be analyzed in the environmental samples, including dust, soil, and water (the full list is provided in Multimedia Appendix 3). The corresponding metabolites, commonly monitored in biomonitoring studies, will be measured in urine samples.

#### Analysis of Pesticides in Liquid Samples (Drinking Water and Urine)

Liquid samples will undergo solid-phase extraction to concentrate and isolate the target pesticides. The extracted samples will then be analyzed via ultraperformance liquid chromatography–triple quadrupole mass spectrometry using electrospray ionization in both the positive and negative modes.

#### Analysis of Pesticides in Solid Samples (Soil and Dust)

For the analysis of soil samples near the households, the soil will be first sieved to 2 mm and dried for 24 hours. For the analysis of indoor dust samples, a wipe will be used to collect the dust accumulated in the petri dish. For floor surface samples, the premoistened wipes will not undergo any special preparation before extraction. All wipes and soil samples will then be extracted via the QUECHERS (quick, easy, cheap, effective, rugged, and safe) method based on the EN 15662:2008 standard with some minor modifications. This method uses an acidified acetonitrile-water mixture followed by a salting out–induced phase separation using a mixture of inorganic salts (NaCl and MgSO_4_) and a citrate buffer. The extract is further purified using dispersive solid-phase extraction and analyzed via ultraperformance liquid chromatography–triple quadrupole mass spectrometry using electrospray ionization in both the positive and negative modes.

### Data Management and Analysis

#### Data Management

Data from the EPURA study will be entered into a Microsoft Access database using a double data entry procedure to ensure accuracy and minimize errors. The database is hosted on a secure Institut national de la recherche scientifique server, with access restricted to authorized research staff, and all information will remain pseudonymized (see the Ethical Considerations section).

#### Data Analysis

Our statistical analyses will include several comparisons of pesticide concentrations in urine. First, we will compare urinary concentrations by subgroups (eg, children vs adults, men vs women, various socioeconomic groups, and farmers vs nonfarmers) to identify potential subgroups presenting higher pesticide exposure. Additionally, we will compare urinary pesticide levels between the growing season (ie, summer) and winter for each participant to estimate differences likely attributable to local pesticide applications. Finally, pesticide levels in urine will be compared with data from the Canadian Health Measures Survey conducted by Health Canada and the Public Health Agency of Canada. This survey program includes measures of several pesticides and metabolites in a representative sample of the Canadian population [33]. When relevant, measured concentrations will further be evaluated against available biomonitoring equivalents and other health-based guidance values to facilitate the interpretation of exposure in relation to potential health risks [34].

Where possible, we will apply a reverse dosimetry approach using pharmacokinetic models to estimate the exposure dose of each participant (in milligrams per day per kilogram of body weight) based on urinary pesticide concentrations. Additionally, we will calculate pesticide intake from water and food to assess their respective contributions to the internal dose both in winter and in summer.

We will model the influence of field proximity and agricultural intensity surrogates on pesticide presence or concentrations in urine and environmental samples. Additionally, we will examine household-related factors (eg, composition and presence of pets), residential characteristics (eg, ventilation and water treatment), and occupant characteristics and lifestyles (eg, pesticide use, airing and cleaning habits, time spent indoors and outdoors, and activities) that could affect pesticide concentrations. We will also investigate gender-related differences, such as dietary patterns linked to pesticide exposure doses. Our models will account for the hierarchical and dependency structure of the data when applicable (eg, multiple measurements per individual and multiple individuals per household) using a sophisticated mixed model [35]. To identify the most relevant determinants, high-dimensional variable selection methods adapted to censored environmental data, such as convex conditioned least absolute shrinkage and selection operator and direct sparse regression procedure using covariance from multi-modality data, will be applied [36,37].

### Ethical Considerations

#### Ethics Approval of the Protocol

The EPURA study protocol received institutional ethics approval from the Institut national de la recherche scientifique in April 2023 prior to participant recruitment and data collection.

#### Consent of the Participants

During the initial meeting with a research assistant, participants provided written informed consent signed by individuals aged 14 years and older, as well as written assent from minors or verbal assent if they were unable to sign but capable of understanding the nature of the project. Participants had the right to withdraw their consent at any time without any negative consequences.

#### Confidentiality and Personal Data Management

During data management and analyses, all personal information will be pseudonymized by replacing participant names with unique identifying codes, and all data will be securely stored, accessible only to authorized researchers. The data will be stored for 10 years. The biological material (urine) that has not been analyzed will be destroyed 10 years after the end of the study. Identifiable information, along with biological and environmental samples, will be destroyed upon completion of the study.

#### Dissemination of Results

Each participant received compensation of CAD $50 (CAD $1=US $0.71 as of July 22, 2026) upon completing their participation in the study. Additionally, a draw for a reward worth CAD $750 was held each year of recruitment for participants who completed all study requirements. Each participant will receive a simplified report summarizing the study’s main outcomes and a personalized report detailing the pesticide concentrations measured in their own samples accompanied by clear, accessible information to help them understand and interpret these results. Public information sessions may also be offered to present the study’s overall findings.

## Results

The EPURA project was funded in 2023 for a five-year period. As of June 1, 2026, participant recruitment and data collection for the EPURA study are complete. During the first study year (2024-2025), 2 municipalities meeting the eligibility criteria were selected for participation. A total of 78 households, corresponding to 178 participants, were enrolled, representing 2.8% (178/6290) of the municipalities’ population. To achieve the recruitment target, 2 additional municipalities were included during the second study year (2025-2026). An additional 95 households, corresponding to 194 participants, were recruited. Overall, the study enrolled 173 households and 372 participants across the 4 selected municipalities, representing an overall participation rate of 2% (372/17,893).

Summer 2024 data collection included 267 wipe samples, 77 petri dish samples, 65 water samples, 78 yard soil samples, and 600 urine samples, whereas winter 2025 data collection included 202 wipe samples and 473 urine samples. Summer 2025 data collection included 336 wipe samples, 93 petri dish samples, 92 water samples, 92 yard soil samples, and 686 urine samples. In winter 2026, a total of 244 wipe samples and 498 urine samples were collected.

Laboratory analyses of environmental and urine samples are being conducted progressively. Environmental samples collected during the first study year, including petri dish dust and soil samples, have been analyzed. Completion of all laboratory analyses is expected in 2027, after which the statistical analyses described in the Methods section will be conducted in accordance with the study objectives. The first publications are expected in 2027, and the study is expected to generate scientific publications for several years thereafter.

## Discussion

### Perspectives

It is realistically expected that the use of synthetic pesticides in Quebec will continue for many years and even increase despite efforts to find safer, effective, and affordable alternatives. Predictive models suggest that climate change will exacerbate plant health issues caused by insects, diseases, and weeds, which may further drive pesticide use [38]. Additionally, Quebec’s goal of enhancing food autonomy is expected to expand agricultural activities, emphasizing the importance of preventing risks to the agricultural communities.

The EPURA study offers a comprehensive approach to addressing these challenges. It will provide crucial insights by quantifying pesticide exposure levels, identifying exposed populations, and characterizing the factors influencing exposure. These findings will serve as a foundation for developing targeted strategies and interventions to minimize potential health risks associated with pesticide exposure. The study findings may also inform discussions on pesticide use regulations, buffer zones, and pesticide drift mitigation strategies, as well as support the development of preventive recommendations for individuals living near agricultural fields, including guidance on hygiene practices and water treatment.

### Strengths and Limitations

This pioneer study presents several strengths. It uses an integrated, multi-matrix approach measuring pesticides in biological and environmental matrices, a comprehensive methodology that has previously been rarely applied at this scale. The study’s large sample size and extended multi-season data collection period enhance reliability and validity, offering nearly a full year of participation for each household.

Most studies investigating pesticide exposure in agricultural communities have relied either on environmental sampling, most commonly house dust, or on biological monitoring such as urine [5]. Only a limited number of studies have combined personal and environmental matrices, with sampling strategies that did not allow for the identification of exposure sources [39-41]. To our knowledge, the PestiRiv study conducted in vineyard areas in France is among the few studies to have assessed a broad range of exposure sources and matrices, including ambient and indoor air, house dust, urine, hair, and self-produced food [42]. Similarly, the OBO study, conducted in a bulb-growing agricultural context in the Netherlands, combined personal samples (urine and hand wipes) with environmental measurements (air, dust, and soil) but focused exclusively on nondietary exposure pathways [21]. The EPURA study builds on these previous initiatives by extending the investigation of pesticide exposure to vegetable-producing areas, an agricultural setting that has so far received limited attention. The study also provides a more comprehensive characterization of individual exposure through repeated urinary measurements collected over the growing season (4 months). In contrast, previous studies have generally relied on much shorter monitoring periods, ranging from a single day in the OBO study to 14 days in the PestiRiv study. Finally, unlike the OBO study, which focused exclusively on nondietary exposure pathways [21], the EPURA study evaluates both dietary and nondietary sources of exposure. This is achieved through the collection of repeated urinary measurements; detailed dietary information; and environmental samples from matrices that have rarely been investigated in residential exposure studies, including wipe and petri dish dust, yard soil, and drinking water.

Furthermore, as the first study of its kind in Canada, the EPURA study will generate original, wide-ranging insights into the public health implications of pesticide exposure among individuals living near areas of agricultural pesticide application.

However, some limitations must be acknowledged. The lack of detailed data on pesticide use limits the ability to directly link agricultural practices to community exposure. The protocol’s high level of participant involvement may introduce selection bias, potentially favoring highly motivated or more educated individuals. However, because the study aims primarily to investigate exposure pathways and determinants rather than estimate exposure prevalence in a reference population, the impact of selection bias on the associations under investigation is expected to be limited. In addition, the extensive sampling protocol and repeated data collection may increase participant burden, potentially leading to attrition or incomplete data collection over the follow-up period. Additionally, as with similar studies, analytical constraints such as high limits of detection for certain pesticides may reduce data completeness in some matrices. Finally, Quebec has specific climatic conditions, agricultural practices, and population characteristics. Extrapolation of the findings of this study to other agricultural contexts should therefore be undertaken with caution. Levels of pesticide exposure and associated sources may vary across regions in Canada.

### Conclusions

Despite these challenges, the EPURA study provides an important basis for improving our understanding of pesticide exposure in agricultural communities and supports efforts to develop evidence-based interventions and public health policies aimed at reducing exposure and associated risks.

## Data Availability

The datasets generated or analyzed during this study are not publicly available or shared with third parties, in accordance with the ethics approval governing this study and the terms of the informed consent form signed by participants.

## Appendix

Multimedia Appendix 1 Self-administered questionnaires.

Multimedia Appendix 2 Household questionnaire.

Multimedia Appendix 3 List of target pesticides.

Multimedia Appendix 4 Research ethics approval letter.

Multimedia Appendix 5 Peer Review Report by the Public, Community & Population Health Review Committee, Canadian Institutes of Health Research (CIHR).

## Abbreviations

EPURA: Exposure to Pesticides Used in Regions of Agriculture
