# Supplementary material for: A Multi-Pathway Approach to Pesticide Exposure in Agricultural Communities: Protocol for an Observational Study
Source: JMIR Res Protoc. 2026 Aug 21;15:e94740. doi: 10.2196/94740 (PMC13545533; doi:10.2196/94740)
Supplement: Supplementary file 1 [file resprot_v15i1e94740_app1.pdf]

QUESTIONNAIRE INDIVIDUEL - Adultes et adolescents résidents

Date de remplissage du questionnaire : ____ / ____ / _________
JJ MM AAAA

A. PRÉLÈVEMENTS

1. Avez-vous bien recueilli votre urine aujourd'hui?
☐ Oui ☐ Non ☐ Ne sait pas

SI OUI :

- **S'agissait-il des premières urines collectées au réveil?** ☐ Oui (1) ☐ Non (0)
- **Depuis combien de temps n'aviez-vous pas uriné ?** *Si vous ne connaissez pas la durée exacte, indiquez une durée approximative.*
  ________heures ________minutes ou ☐ Ne sait pas
- **Avez-vous placé immédiatement le pot au congélateur ?** ☐ Oui (1) ☐ Non (0)
- **Quels sont votre poids et votre taille ?**
  Poids : |__||__||__| , |__| kg ou |__||__||__| lbs ☐ Ne sait pas
  Taille : |__||__||__|cm ou |__|pieds|__||__|pouces ☐ Ne sait pas

2. Avez-vous essuyé le sol de votre entrée avec une lingette ce matin ?
☐ Oui (1) ☐ Non (0) ☐ Ne sait pas (98)

SI OUI :

- **Avez-vous placé le prélèvement au congélateur, séparément des pots d'urines ?** ☐ Oui (1) ☐ Non (0)
- **Quand ce sol avait-t-il été nettoyé pour la dernière fois ?**
  ☐ Il y a moins d'une semaine (0) ☐ Il y a plus d'une semaine (1) ☐ Ne sait pas (98)
- **Avec quoi ce sol avait-il été nettoyé la dernière fois ?**
  ☐ un balai, un porte-poussière (0) ☐ une moppe/vadrouille ou lingette humide (1)
  ☐ un aspirateur, une balayeuse (2) ☐ ne sait pas (98)

B. VOS ACTIVITÉS

3. Hier (de minuit à minuit), combien de temps avez-vous passé dans chacun des lieux suivants ?
Si vous ne connaissez pas la durée exacte, indiquez une durée approximative. Si vous n'avez pas fréquenté un de ces lieux, indiquez 00heures 00minutes.

A l'intérieur de votre domicile	________heures ________minutes
Dehors, dans votre cour ou votre jardin	________heures ________minutes
Dehors, dans un lieu situé dans votre municipalité	________heures ________minutes

4. Avez-vous fait une de ces activités <u>hier (de minuit à minuit)</u> chez vous ?

	**OUI** (1)	**NON** (0)	**NE SAIT PAS** (98)
Du jardinage (entretien de la pelouse, plantation de fleurs et légumes, bêchage, etc.)	☐	☐	☐
Des activités physiques dans votre cour (jeux, sports, …)	☐	☐	☐
Des activités calmes dans votre cour (repas, lecture, repos, …)	☐	☐	☐
Le ménage à l'intérieur de la résidence	☐	☐	☐

5. <u>Au cours de la semaine précédente</u>, à quelle fréquence avez-vous fait les activités suivantes chez vous ?

	**Jamais** (0)	**Un jour dans la semaine** (1)	**2 à 6 jours dans la semaine** (2)	**Tous les jours** (3)
Du jardinage (entretien de la pelouse, plantation de fleurs et légumes, bêchage, etc.)	☐	☐	☐	☐
Des activités physiques dans votre cour (jeux, sports, …)	☐	☐	☐	☐
Des activités calmes dans votre cour (repas, lecture, repos, …)	☐	☐	☐	☐
Le ménage à l'intérieur du domicile	☐	☐	☐	☐

6. <u>Au cours de la semaine précédente</u>, à quelle fréquence les portes et fenêtres de votre logement ont été ouvertes pour **aérer durant la <u>journée</u>** ?

☐ Jamais (0)
☐ Un jour dans la semaine (1)
☐ Deux à six jours dans la semaine (2)
☐ Tous les jours de la semaine (3)

7. <u>Au cours de la semaine précédente</u>, à quelle fréquence les portes et fenêtres de votre logement ont été ouvertes pour **aérer la <u>nuit</u>**?

☐ Jamais (0)
☐ Un jour dans la semaine (1)
☐ Deux à six jours dans la semaine (2)
☐ Tous les jours de la semaine (3)

C. VOS CONSOMMATIONS AU COURS DES DERNIÈRES 24H

8. Hier <u>(de minuit à minuit)</u>, combien de cigarettes avez-vous fumées ? |__||__| cigarette(s)

D. ALIMENTATION AU COURS DES DERNIÈRES 24H

9. Pour les **<u>ALIMENTS ET BOISSONS que vous avez consommés hier (de minuit à minuit)</u>**, précisez l'origine et les modes préparation dans le tableau suivant.

ALIMENTS	Ces aliments étaient-ils issus de l'agriculture biologique ? *Une réponse possible*				D'où provenaient de ces aliments ? *Plusieurs réponses possibles*			Comment ces aliments ont-ils été préparés avant consommation? *Plusieurs réponses possibles*		
	Non (0)	Oui, en partie (1)	Oui, en totalité (2)	Je ne sais pas (98)	Votre jardin potager	Circuits locaux *(Voisins, marché de producteurs)*	Autres *(supermarché, restauration extérieure…)*	Vous les avez rincés ou trempés	Vous les avez épluchés, enlevé les feuilles extérieures	Vous les avez cuits
**LÉGUMES**										
**Légumes-racines et tubercules** *(pomme de terre, betterave, carotte, navet …)*	☐	☐	☐	☐	☐	☐	☐	☐	☐	☐
**Légumes-bulbes** *(ail, oignon, poireau)*	☐	☐	☐	☐	☐	☐	☐	☐	☐	☐
**Légumes-feuilles** *(laitue, choux, épinard …)*	☐	☐	☐	☐	☐	☐	☐	☐	☐	☐
**Graines vertes ou sèches de légumineuses** (*Haricot, pois)*	☐	☐	☐	☐	☐	☐	☐	☐	☐	☐
**Légumes-fruit** *(aubergine, poivron, tomate, …)*	☐	☐	☐	☐	☐	☐	☐	☐	☐	☐
**Cucurbitacées** *(concombre, melon, courgette, courge)*	☐	☐	☐	☐	☐	☐	☐	☐	☐	☐
**FRUITS**										
**Agrumes** *(orange, clémentine, pamplemousse, citron…)*	☐	☐	☐	☐	☐	☐	☐	☐	☐	☐
**Fruits à pépins** *(pomme, poire)*	☐	☐	☐	☐	☐	☐	☐	☐	☐	☐
**Fruits à noyau** *(abricot, cerise, nectarine, pêche, prune, …)*	☐	☐	☐	☐	☐	☐	☐	☐	☐	☐
**Petits fruits** *(mûre, bleuet, canneberge, raisin, kiwi, framboise, fraise)*	☐	☐	☐	☐	☐	☐	☐	☐	☐	☐
**Fruits à coque** *(amande, noix, noisette, pacane …)*	☐	☐	☐	☐	☐	☐	☐	☐	☐	☐
**Fruits tropicaux** *(avocat, banane, ananas, litchi, mangue, dattes, olives)*	☐	☐	☐	☐	☐	☐	☐	☐	☐	☐

ALIMENTS	Ces aliments étaient-ils issus de l'agriculture biologique ? *Une réponse possible*				D'où provenaient de ces aliments ? *Plusieurs réponses possibles*		
	Non (0)	Oui, en partie (1)	Oui, en totalité (2)	Je ne sais pas (98)	Votre jardin potager	Circuits locaux *(voisins, marché de producteurs)*	Autres *(supermarché, restauration extérieure…)*
**VIANDES, POISSONS, ŒUFS, PRODUITS LAITIERS**							
Œuf	☐	☐	☐	☐	☐	☐	☐
Viande, charcuterie	☐	☐	☐	☐	☐	☐	☐
Poissons et crustacés	☐	☐	☐	☐	☐	☐	☐
Produits laitiers (lait, fromage, yogourt, etc)	☐	☐	☐	☐	☐	☐	☐
**PRODUITS CÉRÉALIERS, HUILES**							
Céréales *(maïs, orge, riz, blé)*	☐	☐	☐	☐	☐	☐	☐
Pain, biscuits et gâteaux	☐	☐	☐	☐	☐	☐	☐
Huiles végétales	☐	☐	☐	☐	☐	☐	☐
**BOISSONS**							
Jus de fruits	☐	☐	☐	☐	☐	☐	☐
Café, thé, infusion, tisanes	☐	☐	☐	☐	☐	☐	☐
Soda (coke, pepsi, 7up, etc), limonade, eaux aromatisées, thé glacé etc.	☐	☐	☐	☐	☐	☐	☐
Autres boissons non alcoolisées *(laits végétaux etc.)*	☐	☐	☐	☐	☐	☐	☐
Boissons alcoolisées *(vin, bière, cidre, digestifs, …)*	☐	☐	☐	☐	☐	☐	☐

10. <u>Hier (de minuit à minuit)</u>, avez-vous bu de l'eau du robinet de votre domicile, y compris dans des breuvages, du thé ou du café (inclure l'eau provenant d'une carafe de type Brita®) ? *Les eaux de cuisson ne sont pas prises en compte.*

☐ Oui (1) ☐ Non (0) ☐ Ne sait pas (98)

11. SI OUI, combien de tasses d'eau du robinet (250 mL) avez-vous bu environ?

|__||__|tasses ☐ Ne sait pas

E. UTILISATION DE PESTICIDES AU COURS DE LA SEMAINE DERNIÈRE

12. <u>Au cours de la semaine dernière</u>, avez-vous appliqué sur vous-même des **produits antimoustiques**, un **shampoing antipoux,** un traitement contre la gale ?

☐ Oui (1) ☐ Non (0) ☐ Ne sait pas (98)

13. Au cours de la semaine dernière, avez-vous réalisé un **traitement <u>antiparasitaire externe</u> de vos animaux** domestiques (sprays, pipettes, …) ?

☐ Oui (1) ☐ Non (0) ☐ Ne sait pas (98)

14. Au cours de la semaine dernière, des pesticides ont-ils été utilisés **à <u>l'intérieur de votre domicile</u>** (insecticides (insectes volants incluant moustiques, rampants, acariens, …), traitements contre les maladies des plantes (fongicides, …))?

☐ Oui (1) ☐ Non (0) ☐ Ne sait pas (98)

15. Au cours de la semaine dernière, des pesticides ont-ils été utilisés **<u>dans votre cour</u>** pour traiter vos plantes (désherbants, insecticides, traitement contre les maladies des plantes) ou des produits de protection du bois (xylophène par exemple) ?

☐ Oui (1) ☐ Non (0) ☐ Ne sait pas (98)

16. Au cours de la semaine dernière, avez-vous utilisé des **pesticides <u>au travail</u>** (désherbants, insecticides, traitement contre les maladies des plantes, antiparasitaires, produits contre les nuisibles, désinfectants…) ?

☐ Oui (1) ☐ Non (0) ☐ Ne sait pas (98)

<u>SI VOUS AVEZ RÉPONDU OUI À L'UNE OU PLUSIEURS DE CES QUESTIONS</u>, veuillez noter dans le tableau ci-dessous, le nom des produits, les dates d'utilisation et la personne utilisatrice. Si possible conserver les emballages ou une photo du produit jusqu'à la prochaine visite de l'équipe de recherche.

**Nom des produits**	**Dates d'utilisation** (format JJ/MM)	**Personne utilisatrice**
		☐ Vous-même ☐ Quelqu'un d'autre
		☐ Vous-même ☐ Quelqu'un d'autre
		☐ Vous-même ☐ Quelqu'un d'autre
		☐ Vous-même ☐ Quelqu'un d'autre
		☐ Vous-même ☐ Quelqu'un d'autre
		☐ Vous-même ☐ Quelqu'un d'autre
		☐ Vous-même ☐ Quelqu'un d'autre

F. COMMENTAIRES

Si vous avez des commentaires à formuler sur ce questionnaire, indiquez les ici :

QUESTIONNAIRE INDIVIDUEL – Agriculteurs(trices)

Date de remplissage du questionnaire : ____ / ____ / _________
JJ MM AAAA

A. PRÉLÈVEMENTS

1. Avez-vous bien recueilli votre urine aujourd'hui?
☐ Oui ☐ Non ☐ Ne sait pas

SI OUI :
- **S'agissait-il des premières urines collectées au réveil?** ☐ Oui (1) ☐ Non (0)
- **Depuis combien de temps n'aviez-vous pas uriné ?** *Si vous ne connaissez pas la durée exacte, indiquez une durée approximative.*
  ________heures ________minutes ou ☐ Ne sait pas
- **Avez-vous placé immédiatement le pot au congélateur ?** ☐ Oui (1) ☐ Non (0)
- **Quels sont votre poids et votre taille ?**
  Poids : |__||__||__| , |__| kg ou |__||__||__| lbs ☐ Ne sait pas
  Taille : |__||__||__|cm ou |__|pieds|__||__|pouces ☐ Ne sait pas

2. Avez-vous essuyé le sol de votre entrée avec une lingette ce matin ?
☐ Oui (1) ☐ Non (0) ☐ Ne sait pas (98)

SI OUI :
- **Avez-vous placé le prélèvement au congélateur, séparément des pots d'urines ?** ☐ Oui (1) ☐ Non (0)
- **Quand ce sol avait-t-il été nettoyé pour la dernière fois ?**
  ☐ Il y a moins d'une semaine ☐ Il y a plus d'une semaine ☐ Ne sait pas
- **Avec quoi ce sol avait-il été nettoyé la dernière fois ?**
  ☐ un balai, un porte-poussière (0) ☐ une moppe/vadrouille ou lingette humide (1)
  ☐ un aspirateur, une balayeuse (2) ☐ ne sait pas (98)

B. VOS ACTIVITÉS

3. Hier (de minuit à minuit), combien de temps avez-vous passé dans chacun des lieux suivants ?
Si vous ne connaissez pas la durée exacte, indiquez une durée approximative. Si vous n'avez pas fréquenté un de ces lieux, indiquez 00heures 00minutes.

A l'intérieur de votre domicile	________heures ________minutes
Dehors, dans votre cour ou votre jardin	________heures ________minutes
Sur votre lieu de travail	________heures ________minutes

4. Avez-vous fait une de ces activités <u>hier (de minuit à minuit)</u> chez vous ?

	**OUI** (1)	**NON** (0)	**NE SAIT PAS** (98)
Du jardinage (entretien de la pelouse, plantation de fleurs et légumes, bêchage, etc.)	☐	☐	☐
Des activités physiques dans votre cour (jeux, sports, …)	☐	☐	☐
Des activités calmes dans votre cour (repas, lecture, repos, …)	☐	☐	☐
Le ménage à l'intérieur du domicile	☐	☐	☐

5. <u>Au cours de la semaine précédente</u>, à quelle fréquence avez-vous fait les activités suivantes chez vous ?

	**Jamais** (0)	**Un jour dans la semaine** (1)	**2 à 6 jours dans la semaine** (2)	**Tous les jours** (3)
Du jardinage (entretien de la pelouse, plantation de fleurs et légumes, bêchage, etc.)	☐	☐	☐	☐
Des activités physiques dans votre cour (jeux, sports, …)	☐	☐	☐	☐
Des activités calmes dans votre cour (repas, lecture, repos, …)	☐	☐	☐	☐
Le ménage à l'intérieur du domicile	☐	☐	☐	☐

6. <u>Au cours de la semaine précédente</u>, à quelle fréquence les portes et fenêtres de votre logement ont été ouvertes pour **aérer durant la <u>journée</u>** ?

☐ Jamais (0)
☐ Un jour dans la semaine (1)
☐ Deux à six jours dans la semaine (2)
☐ Tous les jours de la semaine (3)

7. <u>Au cours de la semaine précédente</u>, à quelle fréquence les portes et fenêtres de votre logement ont été ouvertes pour **aérer la <u>nuit</u>**?

☐ Jamais (0)
☐ Un jour dans la semaine (1)
☐ Deux à six jours dans la semaine (2)
☐ Tous les jours de la semaine (3)

C. VOS CONSOMMATIONS AU COURS DES DERNIERES 24H

8. Hier <u>(de minuit à minuit)</u>, combien de cigarettes avez-vous fumées ? |__||__| cigarette(s)

D. ALIMENTATION AU COURS DES DERNIERES 24H

9. Pour les **<u>ALIMENTS ET BOISSONS que vous avez consommés hier (de minuit à minuit)</u>**, précisez vos habitudes de préparation et/ou d'achats dans le tableau suivant.

ALIMENTS	Ces aliments étaient-ils issus de l'agriculture biologique ? *Une réponse possible*				D'où provenaient de ces aliments ? *Plusieurs réponses possibles*			Comment ces aliments ont-ils été préparés avant consommation? *Plusieurs réponses possibles*		
	Non (0)	Oui, en partie (1)	Oui, en totalité (2)	Je ne sais pas (98)	Votre jardin potager	Circuits locaux *(Voisins, marché de producteurs)*	Autres *(supermarché, restauration extérieure…)*	Vous les avez rincés ou trempés	Vous les avez épluchés, enlevé les feuilles extérieures	Vous les avez cuits
**LÉGUMES**										
**Légumes-racines et tubercules** *(pomme de terre, betterave, carotte, navet …)*	☐	☐	☐	☐	☐	☐	☐	☐	☐	☐
**Légumes-bulbes** *(ail, oignon, poireau)*	☐	☐	☐	☐	☐	☐	☐	☐	☐	☐
**Légumes-feuilles** *(laitue, choux, épinard …)*	☐	☐	☐	☐	☐	☐	☐	☐	☐	☐
**Graines vertes ou sèches de légumineuses** (*Haricot, pois)*	☐	☐	☐	☐	☐	☐	☐	☐	☐	☐
**Légumes-fruit** *(aubergine, poivron, tomate, …)*	☐	☐	☐	☐	☐	☐	☐	☐	☐	☐
**Cucurbitacées** *(concombre, melon, courgette, courge)*	☐	☐	☐	☐	☐	☐	☐	☐	☐	☐
**FRUITS**										
**Agrumes** *(orange, clémentine, pamplemousse, citron…)*	☐	☐	☐	☐	☐	☐	☐	☐	☐	☐
**Fruits à pépins** *(pomme, poire)*	☐	☐	☐	☐	☐	☐	☐	☐	☐	☐
**Fruits à noyau** *(abricot, cerise, nectarine, pêche, prune, …)*	☐	☐	☐	☐	☐	☐	☐	☐	☐	☐
**Petits fruits** *(mûre, bleuet, canneberge, raisin, kiwi, framboise, fraise)*	☐	☐	☐	☐	☐	☐	☐	☐	☐	☐
**Fruits à coque** *(amande, noix, noisette, pacane …)*	☐	☐	☐	☐	☐	☐	☐	☐	☐	☐
**Fruits tropicaux** *(avocat, banane, ananas, litchi, mangue, dattes, olives)*	☐	☐	☐	☐	☐	☐	☐	☐	☐	☐

ALIMENTS	Ces aliments étaient-ils issus de l'agriculture biologique ? *Une réponse possible*				D'où provenaient de ces aliments ? *Plusieurs réponses possibles*		
	Non (0)	Oui, en partie (1)	Oui, en totalité (2)	Je ne sais pas (98)	Votre jardin potager	Circuits locaux *(voisins, marché de producteurs)*	Autres *(supermarché, restauration extérieure…)*
**VIANDES, POISSONS, ŒUFS, PRODUITS LAITIERS**							
Œuf	☐	☐	☐	☐	☐	☐	☐
Viande, charcuterie	☐	☐	☐	☐	☐	☐	☐
Poissons et crustacés	☐	☐	☐	☐	☐	☐	☐
Produits laitiers (lait, fromage, yogourt, etc)	☐	☐	☐	☐	☐	☐	☐
**PRODUITS CEREALIERS, HUILES**							
Céréales *(maïs, orge, riz, blé)*	☐	☐	☐	☐	☐	☐	☐
Pain, biscuits et gâteaux	☐	☐	☐	☐	☐	☐	☐
Huiles végétales	☐	☐	☐	☐	☐	☐	☐
**BOISSONS**							
Jus de fruits	☐	☐	☐	☐	☐	☐	☐
Café, thé, infusion, tisanes	☐	☐	☐	☐	☐	☐	☐
Soda (coke, pepsi, 7up, etc), limonade, eaux aromatisées, thé glacé etc.	☐	☐	☐	☐	☐	☐	☐
Autres boissons non alcoolisées *(laits végétaux etc.)*	☐	☐	☐	☐	☐	☐	☐
Boissons alcoolisées *(vin, bière, cidre, digestifs, …)*	☐	☐	☐	☐	☐	☐	☐

10. **<u>Hier (de minuit à minuit)</u>, avez-vous bu de l'eau du robinet de votre domicile, y compris dans des breuvages, du thé ou du café (inclure l'eau provenant d'une carafe de type Brita®) ?**
☐ Oui (1) ☐ Non (0) ☐ Ne sait pas (98)

11. **SI OUI, combien de tasses d'eau du robinet (250 mL) avez-vous bu environ?**
|__||__|tasses ☐ Ne sait pas

E. ACTIVITÉS PROFESSIONNELLES

12. **Au cours de <u>la semaine dernière</u>, à quelle fréquence avez-vous réalisé les tâches suivantes ?**

		**Jamais** (0)	**Une fois** (1)	**Plusieurs fois** (2)	**Tous les jours** (3)
**Activités d'élevage**	Soins aux animaux	☐	☐	☐	☐
	Traite	☐	☐	☐	☐
	Traitements insecticides	☐	☐	☐	☐
	Désinfection des locaux	☐	☐	☐	☐
	Désinfection du matériel de traite	☐	☐	☐	☐
	Autres, précisez : ………………………..	☐	☐	☐	☐
**Cultures végétales**	Traitement de semences à la ferme	☐	☐	☐	☐
	Semis ou plantation	☐	☐	☐	☐
	Application de pesticides (herbicides, insecticides ou fongicides)	☐	☐	☐	☐
	Taille ou autres travaux manuels	☐	☐	☐	☐
	Cueillette/Moisson/Récolte	☐	☐	☐	☐
	Foins	☐	☐	☐	☐
	Nettoyage de pulvérisateurs de pesticides	☐	☐	☐	☐
	Grosses réparations sur pulvérisateurs de pesticides	☐	☐	☐	☐
	Autres, précisez : ………………………..	☐	☐	☐	☐

13. **Au cours de la <u>semaine dernière</u>, à quelle fréquence êtes-vous rentré(e) dans votre domicile avec les vêtements portés sur votre lieu de travail ?**
☐ Jamais (0)
☐ Un jour dans la semaine (1)
☐ Deux à six jours dans la semaine (2)
☐ Tous les jours de la semaine (3)

14. **<u>Au cours de la semaine dernière</u>, à quelle fréquence êtes-vous rentré(e) dans votre domicile avec les chaussures portées sur votre lieu de travail ?**
☐ Jamais (0)
☐ Un jour dans la semaine (1)
☐ Deux à six jours dans la semaine (2)
☐ Tous les jours de la semaine (3)

F. UTILISATION DE PESTICIDES AU COURS DE LA SEMAINE PRÉCÉDENTE

<u>UTILISATIONS DOMESTIQUES</u>

15. **<u>Au cours de la semaine dernière</u>**, avez-vous appliqué sur vous-même des **antimoustiques**, un **shampoing antipoux,** un traitement contre la gale ?
☐ Oui (1) ☐ Non (0) ☐ Ne sait pas (98)

16. **Au cours de la semaine dernière**, avez-vous réalisé un **traitement <u>antiparasitaire externe</u> de vos animaux** domestiques (sprays, pipettes, …) ?
☐ Oui (1) ☐ Non (0) ☐ Ne sait pas (98)

17. **Au cours de la semaine dernière**, des pesticides ont-ils été utilisés **à <u>l'intérieur de votre domicile</u>** (insecticides (insectes volants, rampants, acariens, …), traitements contre les maladies des plantes (fongicides, …))?
☐ Oui (1) ☐ Non (0) ☐ Ne sait pas (98)

18. **Au cours de la semaine dernière**, des pesticides ont-ils été utilisés **<u>dans votre cour</u>** pour traiter vos plantes (désherbants, insecticides, traitement contre les maladies des plantes) ou des produits de protection du bois (xylophène par exemple) ?
☐ Oui (1) ☐ Non (0) ☐ Ne sait pas (98)

<u>SI VOUS AVEZ RÉPONDU OUI À L'UNE OU PLUSIEURS DE CES QUESTIONS</u>, veuillez noter dans le tableau ci-dessous, le nom des produits, les dates d'utilisation et la personne utilisatrice. Si possible conserver les emballages ou une photo du produit jusqu'à la prochaine visite de l'équipe de recherche.

**Nom des produits**	**Dates d'utilisation** (format JJ/MM)	**Personne utilisatrice**
		☐ Vous-même ☐ Quelqu'un d'autre
		☐ Vous-même ☐ Quelqu'un d'autre
		☐ Vous-même ☐ Quelqu'un d'autre
		☐ Vous-même ☐ Quelqu'un d'autre
		☐ Vous-même ☐ Quelqu'un d'autre
		☐ Vous-même ☐ Quelqu'un d'autre
		☐ Vous-même ☐ Quelqu'un d'autre

UTILISATIONS PROFESSIONNELLES

19. Au cours de la semaine dernière, **avez-vous utilisé vous-même des pesticides au travail** (herbicides, insecticides, fongicides, antiparasitaires, produits contre les nuisibles, désinfectants…) ?
☐ Oui (1) ☐ Non (0) ☐ Ne sait pas (98)

SI VOUS AVEZ RÉPONDU OUI À L'UNE OU PLUSIEURS DE CES QUESTIONS, veuillez noter dans le tableau ci-dessous, le nom des produits, les dates d'utilisation et la personne utilisatrice. Si possible conserver les emballages ou une photo du produit jusqu'à la prochaine visite de l'équipe de recherche.

Nom des produits	Dates d'utilisation (format JJ/MM)

20. Si vous avez utilisé des pesticides au travail, portiez-vous certains des équipements de protection suivant?
Des gants : ☐ Oui, toujours (2) ☐ Oui, parfois (1) ☐ Non (0) ☐ Ne sait pas (98)
Une combinaison jetable : ☐ Oui, toujours (2) ☐ Oui, parfois (1) ☐ Non (0) ☐ Ne sait pas (98)
Un masque à cartouche jetable : ☐ Oui, toujours (2) ☐ Oui, parfois (1) ☐ Non (0) ☐ Ne sait pas (98)

G. COMMENTAIRES

Si vous avez des commentaires à formuler sur ce questionnaire, indiquez les ici :

QUESTIONNAIRE INDIVIDUEL – Enfants

Date de remplissage du questionnaire : _____ / _____ / __________
JJ MM AAAA

A. PRELEVEMENTS

1. **Avez-vous bien recueilli l'urine de votre enfant aujourd'hui?** ☐ Oui (1) ☐ Non (0)

   SI OUI
   - **S'agissait-il des premières urines collectées au réveil?** ☐ Oui (1) ☐ Non (0)
   - **Depuis combien de temps votre enfant n'avait pas uriné ?** *Si vous ne connaissez pas la durée exacte, indiquez une durée approximative.* ________heures ________minutes ou ☐ Ne sait pas
   - **Avez-vous placé immédiatement le pot au congélateur ?** ☐ Oui ☐ Non ☐ Ne sait pas
   - **Quels sont le poids et la taille de votre enfant ?**
   - Poids : |__||__||__| **,** |__| kg ou |__||__||__| lbs ☐ Ne sait pas
   - Taille : |__||__||__|cm ou |__|pieds|__||__|pouces ☐ Ne sait pas

B. LES ACTIVITÉS DE VOTRE ENFANT

2. **Hier (de minuit à minuit), combien de temps votre enfant a-t-il passé dans chacun des lieux suivants ?**
   Si vous ne connaissez pas la durée exacte, indiquez une durée approximative. Si votre enfant n'a pas fréquenté un de ces lieux, indiquez 00heures 00minutes.

A l'intérieur de votre domicile	________heures ________minutes
Dehors, dans votre cour ou votre jardin	________heures ________minutes
Dans son lieu de garde ou à l'école s'il est situé dans votre municipalité	________heures ________minutes
Dehors, dans un lieu situé dans votre municipalité	________heures ________minutes

3. **Votre enfant a-t-il fait une de ces activités hier (de minuit à minuit)?**

	OUI (1)	NON (0)	NE SAIT PAS (98)
Du jardinage (entretien de la pelouse, plantation de fleurs et légumes, bêchage, etc.)	☐	☐	☐
Des activités physiques dans votre cour (jeux, sports, …)	☐	☐	☐
Des activités calmes dans votre cour (repas, lecture, repos, …)	☐	☐	☐

4. **Au cours de la semaine précédente, à quelle fréquence votre enfant a-t-il fait les activités suivantes chez vous?**

	Jamais (0)	Un jour dans la semaine (1)	2 à 6 jours dans la semaine (2)	Tous les jours (3)
Du jardinage (entretien de la pelouse, plantation de fleurs et légumes, bêchage, etc.)	☐	☐	☐	☐
Des activités physiques dans votre cour (jeux, sports, …)	☐	☐	☐	☐
Des activités calmes dans votre cour (repas, lecture, repos, …)	☐	☐	☐	☐

C. ALIMENTATION AU COURS DES DERNIERES 24H

5. Pour les **ALIMENTS ET BOISSONS que votre enfant a consommés hier SEULEMENT (de minuit à minuit)**, précisez l'origine et les modes préparation dans le tableau suivant.

ALIMENTS	**Ces aliments étaient-ils issus de l'agriculture biologique ?** *Une réponse possible*				**D'où provenaient de ces aliments ?** *Plusieurs réponses possibles*			**Comment ces aliments ont-ils été préparés avant consommation?** *Plusieurs réponses possibles*		
	Non (0)	Oui, en partie (1)	Oui, en totalité (2)	Je ne sais pas (98)	Votre jardin potager	Circuits locaux *(Voisins, marché de producteurs)*	Autres *(supermarché, restauration extérieure…)*	Vous les avez rincés ou trempés	Vous les avez épluchés, enlevé les feuilles extérieures	Vous les avez cuits
**LÉGUMES**										
**Légumes-racines et tubercules** *(pomme de terre, betterave, carotte, navet …)*	☐	☐	☐	☐	☐	☐	☐	☐	☐	☐
**Légumes-bulbes** *(ail, oignon, poireau)*	☐	☐	☐	☐	☐	☐	☐	☐	☐	☐
**Légumes-feuilles** *(laitue, choux, épinard …)*	☐	☐	☐	☐	☐	☐	☐	☐	☐	☐
**Graines vertes ou sèches de légumineuses** (*Haricot, pois)*	☐	☐	☐	☐	☐	☐	☐	☐	☐	☐
**Légumes-fruit** *(aubergine, poivron, tomate, …)*	☐	☐	☐	☐	☐	☐	☐	☐	☐	☐
**Cucurbitacées** *(concombre, melon, courgette, courge)*	☐	☐	☐	☐	☐	☐	☐	☐	☐	☐
**FRUITS**										
**Agrumes** *(orange, clémentine, pamplemousse, citron…)*	☐	☐	☐	☐	☐	☐	☐	☐	☐	☐
**Fruits à pépins** *(pomme, poire)*	☐	☐	☐	☐	☐	☐	☐	☐	☐	☐
**Fruits à noyau** *(abricot, cerise, nectarine, pêche, prune, …)*	☐	☐	☐	☐	☐	☐	☐	☐	☐	☐
**Petits fruits** *(mûre, bleuet, canneberge, raisin, kiwi, framboise, fraise)*	☐	☐	☐	☐	☐	☐	☐	☐	☐	☐
**Fruits à coque** *(amande, noix, noisette, pacane …)*	☐	☐	☐	☐	☐	☐	☐	☐	☐	☐
**Fruits tropicaux** *(avocat, banane, ananas, litchi, mangue, dattes, olives)*	☐	☐	☐	☐	☐	☐	☐	☐	☐	☐

ALIMENTS	Ces aliments étaient-ils issus de l'agriculture biologique ? *Une réponse possible*				D'où provenaient de ces aliments ? *Plusieurs réponses possibles*		
	Non (0)	Oui, en partie (1)	Oui, en totalité (2)	Je ne sais pas (98)	Votre jardin potager	Circuits locaux *(voisins, marché de producteurs)*	Autres *(supermarché, restauration extérieure…)*
**VIANDES, POISSONS, ŒUFS, PRODUITS LAITIERS**							
Œuf	☐	☐	☐	☐	☐	☐	☐
Viande, charcuterie	☐	☐	☐	☐	☐	☐	☐
Poissons et crustacés	☐	☐	☐	☐	☐	☐	☐
Produits laitiers (lait, fromage, yogourt, etc)	☐	☐	☐	☐	☐	☐	☐
**PRODUITS CEREALIERS, HUILES**							
Céréales *(maïs, orge, riz, blé)*	☐	☐	☐	☐	☐	☐	☐
Pain, biscuits et gâteaux	☐	☐	☐	☐	☐	☐	☐
Huiles végétales	☐	☐	☐	☐	☐	☐	☐
**BOISSONS**							
Jus de fruits	☐	☐	☐	☐	☐	☐	☐
Café, thé, infusion, tisanes	☐	☐	☐	☐	☐	☐	☐
Soda (coke, pepsi, 7up, etc), limonade, eaux aromatisées, thé glacé etc.	☐	☐	☐	☐	☐	☐	☐
Autres boissons non alcoolisées *(laits végétaux etc.)*	☐	☐	☐	☐	☐	☐	☐
Boissons alcoolisées *(vin, bière, cidre, digestifs, …)*	☐	☐	☐	☐	☐	☐	☐

6. **<u>Hier (de minuit à minuit)</u>, votre enfant a-t-il bu de l'eau du robinet du domicile, y compris dans des breuvages (inclure l'eau provenant d'une carafe de type Brita®) ?**
☐ Oui (1) ☐ Non (0) ☐ Ne sait pas (98)

7. **SI OUI, combien de tasses d'eau du robinet (250 mL) a-t-il bu environ?**
|__||__|tasses ☐ Ne sait pas

D. UTILISATION DE PESTICIDES AU COURS DE LA SEMAINE DERNIERE

8. <u>Au cours de la semaine dernière</u>, votre enfant a-t-il reçu des **produits antimoustiques**, un produit **antipoux** ou un traitement contre la gale ?
☐ Oui (1) ☐ Non (0) ☐ Ne sait pas (98)

<u>SI VOUS AVEZ RÉPONDU OUI</u>, pensez à bien noter dans le tableau ci-dessous la date d'utilisation, le nom des produits et si possible conserver les emballages ou une photo des produits jusqu'à la prochaine visite de l'équipe de recherche.

**Nom des produits**	**Dates d'utilisation** (format JJ/MM)

E. COMMENTAIRES

Si vous avez des commentaires à formuler sur ce questionnaire, indiquez les ici :
