# Supplementary material for: A Multi-Pathway Approach to Pesticide Exposure in Agricultural Communities: Protocol for an Observational Study
Source: JMIR Res Protoc. 2026 Aug 21;15:e94740. doi: 10.2196/94740 (PMC13545533; doi:10.2196/94740)
Supplement: Supplementary file 2 [file resprot_v15i1e94740_app2.pdf]

QUESTIONNAIRE MÉNAGE – « ÉTÉ » ($Q_{M\text{-}ete}$)

Nom de l'enquêteur : ________________ **Date de l'enquête** : |__||__|/|__||__|/|__||__||__||__| **Numéro d'identification du ménage :** |__|--|__||__||__|

A. RENSEIGNEMENTS SUR LE MÉNAGE

Les questions suivantes portent sur les **personnes qui habitent <u>habituellement</u> à cette adresse**, qu'ils participent ou non à l'étude.

	Occupants					
	**1**	**2**	**3**	**4**	**5**	**6**
**Numéro d'identification** *(seulement si la personne participe à la collecte d'urine)*						
**1. Quelle est la date de naissance de la personne ?**						
**2. Quel était le sexe à la naissance de cette personne ?**<br>*1 : Masculin*<br>*2 : Féminin*	|__|	|__|	|__|	|__|	|__|	|__|
**<u>SI : ADULTES</u> :**						
**3. Quel est le plus haut certificat, diplôme ou grade que cette personne a obtenu ?**<br>*0 : Niveau inférieur à un diplôme d'études secondaires ou à son équivalent*<br>*1 : Diplôme d'études secondaires ou une attestation d'équivalence d'études secondaires*<br>*2 : Certificat ou diplôme d'une école de métiers*<br>*3 : Certificat ou diplôme d'un collège, d'un cégep ou d'un autre établissement non universitaire (autre que les certificats ou diplômes de métiers)*<br>*4 : Certificat ou diplôme universitaire inférieur au baccalauréat*<br>*5 : Baccalauréat (p. ex. B.A., B. Sc., B. Éd., LL.B.)*<br>*6 : Certificat, diplôme ou grade universitaire supérieur au baccalauréat*	|__|	|__|	|__|	|__|	|__|	|__|

	Occupants					
	1	2	3	4	5	6
**4. L'activité principale de cette personne est-elle de travailler à un emploi ou à une entreprise ?**<br>*0 : Non*<br>*1 : Oui*<br>*98 : Ne sait pas*	|__|	|__|	|__|	|__|	|__|	|__|
**5. SI OUI, quelle est la profession de cette personne ?**						
**6. SI OUI, quel est le secteur d'activité de l'entreprise dans laquelle travaille cette personne ?**<br>*Veiller à bien préciser le type d'exploitation agricole pour le secteur de l'agriculture*						
**7. SI OUI, ce travail implique-t-il la fabrication ou la manipulation de produits pesticides ?**<br>*0 : Non*<br>*1 : Oui*<br>*98 : Ne sait pas*	|__|	|__|	|__|	|__|	|__|	|__|
**8. SINON, quelle est l'activité principale de la personne ?**<br>*0 : Chercher un emploi rémunéré*<br>*1 : Être aux études*<br>*2 : S'occuper de ses vos enfants*<br>*3 : Travaux ménagers*<br>*4 : À la retraite*<br>*5 : Congé de maternité, paternité ou parental*<br>*6 : Maladie de longue durée*<br>*7 : Bénévolat ou des soins à des enfants autres que vos enfants*<br>*8 : Autre*	|__|	|__|	|__|	|__|	|__|	|__|

9. **Au meilleur de votre connaissance, à combien estimez-vous <u>le revenu total de tous les membres résidant à cette adresse</u>, provenant de toutes les sources, avant impôt et autres déductions, au cours de l'année se terminant le 31 décembre ? |__|**
   Le revenu peut provenir de diverses sources comme le travail, les investissements, les pensions ou le gouvernement. Les exemples incluent l'assurance-emploi, l'aide sociale, les prestations fiscales pour enfants et d'autres revenus tels que les pensions alimentaires et les revenus locatifs.

   0: Moins de 10 000 $
   1: 10 001 $ – 20 000 $
   2: 20 001 $ – 30 000 $
   3: 30 001 $ – 40 000 $
   4: 40 001 $ – 50 000 $
   5: 50 001 $ – 60 000 $
   6: 60 001 $ – 70 000 $
   7: 70 001 $ – 80 000 $
   8: 80 001 $ – 100 000 $
   9: 100 001 $ – 150 000 $
   10: Plus de 150 000 $
   98 : Ne sait pas
   99 : Ne souhaite pas répondre

10. **Combien de personnes sont soutenues par ce revenu total (qu'ils habitent ou non à cette adresse) ? |__||__|**

B. RENSEIGNEMENTS SUR LA RÉSIDENCE

11. **De quel type de logement s'agit-il** ? |__|
   1: Maison individuelle
   2 : Immeuble à logements multiples avec entrée privée
   3 : Immeuble à logements multiples avec entrée commune
   4 : Autre (Logement mobile, institution, …)

12. **La résidence est-elle située sur une exploitation agricole ? |__|**
   0 : Non, *1 : Oui,* *98 : Ne sait pas*

   13. **Si oui, de quel type d'exploitation s'agit-il ? |__|**
      0 : Maraîchage
      1: Grandes cultures
      2: Polyculture-Elevage
      3 : Arboriculture/viticulture
      4 : Apiculture
      98 : ne sait pas

   14. **Si oui, s'agit-il d'une exploitation en agriculture biologique ou conventionnelle ? |__|**
      0 : Agriculture biologique, *1: Agriculture conventionnelle,* *98 : Ne sait pas*

15. **Quelle est la surface totale de la résidence (pièces habitables) ? |__||__||__|**m^2 ou |__||__||__||__|pi^2

16. **SINON, à combien l'estimez-vous ? |__|**
   0 : ≤50 m² (≤ 540 pi²)
   1 : 50-99 m² (540 - 1070 pi²)
   2 : 100-149 m² (1070-1610 pi²)
   3 : ≥ 150 m² (≥ 1610 pi²)
   98 : Ne sait pas

17. **Quelle est la période de construction ou de dernière rénovation majeure du bâtiment ? |__|**
   0 : Avant 1976
   1 : 1977 - 1983
   2 : 1984 - 1993
   3 : 1994 - 2000
   4 : 2001 - 2008
   5 : 2008 - 2015
   6 : 2016 - 2021
   7 : 2022 à aujourd'hui
   98 : Ne sait pas

18. Avez-vous effectué des travaux de rénovation dans la résidence au cours des 12 derniers mois ? |__|

0 : Non, *1 : Oui,* *98 : Ne sait pas*

19. Si oui, de quels types ? |__| |__| |__|

0 : charpente/menuiseries extérieures
1 : autres travaux de gros œuvre
2 : isolation phonique et thermique intérieure
3 : pose de revêtements
4 : maçonnerie/plomberie/électricité
5 : menuiseries intérieures
6 : peintures
7 : autres travaux de second-œuvre
8 : tous travaux/construction totale

20. La résidence dispose-t-elle d'un système d'échangeur d'air indépendant <u>distinct</u> du système de chauffage/refroidissement ? |__|

0 : Non *1 : Oui* *98 : Ne sait pas*

21. Dans votre résidence, disposez-vous des systèmes de chauffage / refroidissement suivants ?

0 : Non, *1 : Oui,* *98 : Ne sait pas*

… un climatiseur mural (produit du froid) ? |__|
… une unité de climatiseur portable (produit du froid) ? |__|
… une thermopompe murale (produit du froid et de la chaleur) ? |__|
… un chauffage à air pulsé, une thermopompe centrale, une pompe à chaleur (échange d'air avec l'extérieur)? |__|

22. SI OUI, au cours de l'été, en moyenne, à quelle fréquence avez-vous fait fonctionner votre système de refroidissement ? |__|

0 : Moins d'une fois par mois
1 : Plus d'une fois par mois mais moins d'une fois par semaine
2 : Une fois par semaine
3 : Plusieurs fois par semaine
4 : Tous les jours ou presque

23. Quelle est la source de l'eau du robinet dans ce domicile? |__|

0 : De l'eau fournie par la ville, le village ou la municipalité
1 : De l'eau provenant d'un puits privé
2 : De l'eau provenant d'une citerne
3 : De l'eau provenant d'une source de surface
4 : Autre
98 : Ne sait pas

24. Les occupants du domicile boivent-ils habituellement l'eau du robinet ? |__|

0 : Non *1 : Oui* *98 : Ne sait pas*

25. Comment l'eau utilisée comme boisson à ce domicile est-elle traitée? |__||__||__|

0 : Un filtre (p. ex. filtre attaché à un robinet, un filtre-réfrigérateur, carafe filtrante type Brita®)
1 : Un adoucisseur d'eau
2 : Stérilisation par rayons ultra-violets
3 : L'osmose inversée
4 : L'ébullition
5 : La distillation
6 : Autre
7 : Pas de traitement
98 : Ne sait pas

26. Comment l'eau utilisée pour la préparation des repas à ce domicile est-elle traitée? |__||__||__|

0 : Un filtre (p. ex. filtre attaché à un robinet, filtre réfrigérateur, carafe filtrante type Brita®)
1 : Un adoucisseur d'eau
2 : Stérilisation par rayons ultra-violets
3 : L'osmose inversée
4 : L'ébullition
5 : La distillation
6 : Autre
7 : Pas de traitement
98 : Ne sait pas

C. PRATIQUES D'HYGIÈNE AU DOMICILE

Au cours de l'été, <u>en moyenne</u>, …

27. … à quelle fréquence a été passée la moppe dans votre résidence ? |__|

0 : Moins d'une fois par mois
1 : Plus d'une fois par mois mais moins d'une fois par semaine
2 : Une fois par semaine
3 : Plusieurs fois par semaine
4 : Tous les jours ou presque

28. … à quelle fréquence a été passée l'aspirateur/la balayeuse dans votre résidence ? |__|

0 : Moins d'une fois par mois
1 : Plus d'une fois par mois mais moins d'une fois par semaine
2 : Une fois par semaine
3 : Plusieurs fois par semaine
4 : Tous les jours ou presque

29. … à quelle fréquence a été passée le balai dans votre logement ? |__|

0 : Moins d'une fois par mois
1 : Plus d'une fois par mois mais moins d'une fois par semaine
2 : Une fois par semaine
3 : Plusieurs fois par semaine
4 : Tous les jours ou presque

30. … à quelle fréquence avez-vous ouvert vos portes et fenêtres pour aérer en journée ? |__|

0 : Moins d'une fois par mois
1 : Plus d'une fois par mois mais moins d'une fois par semaine
2 : Une fois par semaine
3 : Plusieurs fois par semaine
4 : Tous les jours ou presque

31. … à quelle fréquence avez-vous ouvert vos portes et fenêtres pour aérer la nuit? |__|

0 : Moins d'une fois par mois
1 : Plus d'une fois par mois mais moins d'une fois par semaine
2 : Une fois par semaine
3 : Plusieurs fois par semaine
4 : Tous les jours ou presque

32. … à quelle fréquence les occupants se sont-ils déchaussés dans la résidence ? |__|

0 : Jamais
1 : Occasionnellement
2 : Souvent
3 : Systématiquement

33. … une ou plusieurs personnes fument-elles à l'intérieur de la résidence ? |__|

0 : Non *1 : Oui* *98 : Ne sait pas*

<u>Si un ou plusieurs habitants exercent des emplois impliquant la manipulation/ fabrication de pesticides en moyenne au cours de l'été,</u> …

34. ... à quelle fréquence cette(ces) personne(s) sont-elles rentrées dans le domicile avec les vêtements portés sur leur lieu de travail ? |__|

0 : Moins d'une fois par mois
1 : Plus d'une fois par mois mais moins d'une fois par semaine
2 : Une fois par semaine
3 : Plusieurs fois par semaine
4 : Tous les jours ou presque

35. ... à quelle fréquence cette(ces) personne(s) sont-elles rentrées dans le domicile avec les chaussures portées sur leur lieu de travail ? |__|

0 : Moins d'une fois par mois
1 : Plus d'une fois par mois mais moins d'une fois par semaine

2 : Une fois par semaine *4 : Tous les jours ou presque*
3 : Plusieurs fois par semaine

36. Y-a-t-il des paillassons aux entrées de la résidence ? |__|
0 : Non, 1 : Oui, au niveau de quelques entrées, 2 : Oui, au niveau de toutes les entrées

37. Possédez-vous un ou plusieurs animaux domestiques <u>autorisés à entrer à l'intérieur du domicile</u> ? |__|
0 : Non, 1 : Oui

38. SI OUI, quels types d'animaux possédez-vous ? *Indiquez le nombre.*

Chat : |__| Chien :|__| Autres :|__|, précisez : __________________________

39. SI OUI, au cours de l'été, en moyenne, à quelle fréquence les animaux domestiques sont-ils sortis dans votre cour ou dans l'environnement proche de la résidence ? |__|
0 : Moins d'une fois par mois
1 : Plus d'une fois par mois mais moins d'une fois par semaine
2 : Une fois par semaine
3 : Plusieurs fois par semaine
4 : Tous les jours ou presque

D. UTILISATION DE PESTICIDES À L'INTÉRIEUR DE LA RÉSIDENCE

40. <u>Durant le printemps et l'été</u>, des produits pesticides ont-ils été utilisés par un membre du foyer ou un professionnel à l'intérieur du domicile (antiparasitaires pour animaux, insecticides (insectes volants, rampants, acariens, …), traitements contre les maladies des plantes (fongicides, …))**?**

	Nom commercial du produit utilisé et substance(s) active(s) (si indiqué)	Cible principale*	Combien de fois le produit a été appliqué durant le printemps et l'été?					A quand remonte la dernière utilisation ?			
			1 fois (0)	2 à 5 fois (1)	6 à 9 fois (2)	≥ 10 fois (3)	≥ 10 fois ou en continu (4) **	> 6 mois (0)	6-3 mois (1)	3-1 mois (2)	≤ 1 mois (3)
**1.**		|__|	☐	☐	☐	☐	☐	☐	☐	☐	☐
**2.**		|__|	☐	☐	☐	☐	☐	☐	☐	☐	☐
**3.**		|__|	☐	☐	☐	☐	☐	☐	☐	☐	☐
**4.**		|__|	☐	☐	☐	☐	☐	☐	☐	☐	☐
**5.**		|__|	☐	☐	☐	☐	☐	☐	☐	☐	☐

**Cible : 0 : insectes rampants, 1 : insectes volants, 2 : mollusque, 3 : moisissures, les champignons, algues, 4 : mauvaises herbes, 5 : maladies des plantes, 6 : antiparasitaires pour animaux, 7 : antiparasitaires pour humain, 8 : ravageurs du bois, 9 : rongeurs et taupes, 10 : autres*

*** : « en continu » pour les équipements diffusant en continu sur plusieurs semaines, de type colliers antipuces, boîtes anti-fourmis, autocollants anti-mouches*

E. COUR EXTÉRIEURE

41. La résidence possède-t-elle un espace extérieur ? |__|

0 : Non, *1 : Oui*

42. SI OUI, possédez-vous ?

0 : Non, *1 : Oui*

… un jardin potager : |__|

… des arbres fruitiers : |__|

… un jardin d'agrément, une terrasse : |__|

… un clapier ou un poulailler : |__|

<u>SI OUI</u>, au cours de l'été, en moyenne …

43. …. à quelle fréquence avez-vous séché votre linge dans votre cour ? |__|

0 : Moins d'une fois par mois
1 : Plus d'une fois par mois mais moins d'une fois par semaine
2 : Une fois par semaine
3 : Plusieurs fois par semaine
4 : Tous les jours ou presque

44. … à quelle fréquence les occupants de la résidence ont-ils jardiné ? |__|

0 : Moins d'une fois par mois
1 : Plus d'une fois par mois mais moins d'une fois par semaine
2 : Une fois par semaine
3 : Plusieurs fois par semaine
4 : Tous les jours ou presque

45. … à quelle fréquence les occupants de la résidence ont-ils pratiqué des activités physiques dans la cour (jeux, sports) ? |__|

0 : Moins d'une fois par mois
1 : Plus d'une fois par mois mais moins d'une fois par semaine
2 : Une fois par semaine
3 : Plusieurs fois par semaine
4 : Tous les jours ou presque

46. … à quelle fréquence les occupants de la résidence ont-ils pratiqué des activités calmes dans la cour (repas, lecture, repos, …) ? |__|

0 : Moins d'une fois par mois
1 : Plus d'une fois par mois mais moins d'une fois par semaine
2 : Une fois par semaine
3 : Plusieurs fois par semaine
4 : Tous les jours ou presque

47. Votre résidence a-t-elle été traitée contre les insectes qui attaquant le bois (fourmis charpentières, lyctes, longicornes, vrillette …) au cours des 5 dernières années ? |__|

0 : Non *1 : Oui* *98 : Ne sait pas*

48. SI OUI, en quelle année ? |__||__||__|__|

49. SI OUI, quel produit a été utilisé (nom commercial ou substance) ?

----

50. <u>Durant le printemps et l'été</u>, certains produits pesticides ont-ils été utilisés par un membre du foyer ou un professionnel à l'extérieur pour traiter vos plantes (désherbants, insecticides, traitement contre les maladies des plantes) **ou les animaux** (antiparasitaires) **ou des produits de protection du bois** (xylophène par exemple) **? A quelle fréquence ? |__|**

	Nom commercial du produit utilisé et substance(s) active(s) (si indiqué)	Cible principale*	Combien de fois le produit a été appliqué durant le printemps et l'été?					A quand remonte la dernière utilisation ?			
			1 fois (0)	2 à 5 fois (1)	6 à 9 fois (2)	≥ 10 fois (3)	≥ 10 fois ou en continu (4) **	> 6 mois (0)	6-3 mois (1)	3-1 mois (2)	≤ 1 mois (3)
**1.**		|__|	☐	☐	☐	☐	☐	☐	☐	☐	☐
**2.**		|__|	☐	☐	☐	☐	☐	☐	☐	☐	☐
**3.**		|__|	☐	☐	☐	☐	☐	☐	☐	☐	☐
**4.**		|__|	☐	☐	☐	☐	☐	☐	☐	☐	☐
**5.**		|__|	☐	☐	☐	☐	☐	☐	☐	☐	☐

**Cible : 0 : insectes rampants, 1 : insectes volants, 2 : mollusques, 3 : moisissures, champignons, algues, 4 : mauvaises herbes, 5 : maladies des plantes, 6 : antiparasitaires pour animaux, 7 : antiparasitaires pour humain, 8 : ravageurs du bois, 9 : rongeurs et taupes, 10 : autres*
*** : « en continu » pour les équipements diffusant en continu sur plusieurs semaines, de type colliers antipuces, boîtes anti-fourmis, autocollants anti-mouche*
