# Supplementary material for: A Multi-Pathway Approach to Pesticide Exposure in Agricultural Communities: Protocol for an Observational Study
Source: JMIR Res Protoc. 2026 Aug 21;15:e94740. doi: 10.2196/94740 (PMC13545533; doi:10.2196/94740)
Supplement: Supplementary file 3 [file resprot_v15i1e94740_app3.pdf]

Fongicides (n=18)

ametoctradin, azoxystrobin, boscalid, cyazofamid, cyprodinil, difenoconazole, (E)-dimethomorph, (Z)-dimethomorph, fenamidone, fludioxonil, fluopicolide, fluoxastrobin, fluxapyroxad, mandipropamid, metalaxyl, pyraclostrobin, pyrimethanil, trifloxystrobin

Herbicides (n=19)

2,4-dichlorophenoxyacetic acid (2,4-D), (4-chloro-2-methylphenoxy)acetic acid (MCPA), atrazine, bentazone, bromoxynil, (E)-clethodim, (Z)-clethodim, clopyralid, dicamba, dimethenamid-P, fluazifop-P-Butyl, flumioxazin, fomesafen, linuron, mesotrione, metolachlor, oxyfluorfen, pendimethalin, prometryn

Insecticides (n=14)

abamectin B1a, acetamiprid, carbaryl, chlorantraniliprole, clothianidin, cyantraniliprole, cypermethrin, deltamethrin, dimethoate, flonicamid, imidacloprid, malathion, cis-permethrin et trans-permethrin
