# Supplementary material for: A Multi-Pathway Approach to Pesticide Exposure in Agricultural Communities: Protocol for an Observational Study
Source: JMIR Res Protoc. 2026 Aug 21;15:e94740. doi: 10.2196/94740 (PMC13545533; doi:10.2196/94740)
Supplement: Supplementary file 4 [file resprot_v15i1e94740_app4.pdf]

Le 03 avril 2024

À l'attention de :
Maryse Bouchard, Professeur(e)
Centre Armand Frappier Santé-Biotechnologie

Titre : Exposition aux pesticides des communautés vivant à proximité de sites de production agricole intensive : Quantifier les sources d'exposition pour agir en prévention
# de projet : 2024-253, 24-767

Objet : Approbation éthique de votre projet de recherche

Bonjour,

En réponse à votre demande de certificat éthique, pour le projet de recherche mentionné en titre et à la suite de l'évaluation effectuée par le Comité d'éthique en recherche avec des être humains de l'INRS (CER-INRS), nous avons le plaisir de vous confirmer l'acceptation de votre demande, de vos réponses aux conditions et l'émission du certificat. Ce dernier, émis en date du 03 avril 2024 est joint à la présente communication.

Votre certificat est valide jusqu'au **03 avril 2025**.

Avant qu'il soit échu, vous recevrez une notification de Nagano (selon vos options de notifications) et le formulaire de suivi (F7) apparaîtra dans votre tableau de bord de la plateforme Nagano, sous "projet en renouvellement". Les chercheuses et chercheurs qui ne respecteront pas l'obligation de compléter le formulaire de renouvellement verront leur certificat d'éthique suspendu, ce qui entraînera automatiquement le gel des fonds liés au projet de recherche pour lequel le certificat a été émis.

De plus, il vous est rappelé qu'il est également de votre responsabilité d'informer le comité de toutes **modification significative** (à la population ciblée, au formulaire de consentement, au protocole d'expérimentation, à la méthode de collecte ou de traitement des données, etc.) **ou affectant le niveau de risque du projet,** et ce **AVANT** de les mettre en place.

- Une copie de ce certificat est transmise à l'équipe post-octroi du Service à la recherche de l'INRS, qui pourra faciliter l'accès aux fonds de recherche (le cas échéant), mais il est de votre responsabilité de transmettre votre certificat d'éthique à votre organisme subventionnaire, au besoin.
-
- Dans le cadre de l'Entente pour la reconnaissance des certificats d'éthique des projets de recherche à risque minimal, il est de votre responsabilité d'informer vos co-chercheuses et co-chercheurs d'institutions externe, de l'obtention de votre certification.

Enfin, nous vous demandons d'utiliser pour votre projet les numéros de référence suivants **2024-253, 24-767** pour toute correspondance avec le CER-INRS.

En vous souhaitant le meilleur des succès dans la réalisation de votre recherche, veuillez recevoir nos salutations distinguées.

Le CER-INRS

Pièce jointe: Certificat d'approbation éthique

CERTIFICAT D'APPROBATION ÉTHIQUE

Le Comité d'éthique en recherche avec des êtres humains de l'INRS a examiné le projet de recherche identifié ci-dessous et atteste que la recherche proposée impliquant des êtres humains réponds aux normes de l'INRS en matière d'éthique.

À la lumière de l'évaluation faite :

- le projet satisfait les critères d'une recherche à risque minimal.

Projet : 2024-253, 24-767

Titre du projet de recherche : Exposition aux pesticides des communautés vivant à proximité de sites de production agricole intensive : Quantifier les sources d'exposition pour agir en prévention

Chercheuse ou chercheur responsable

Maryse Bouchard, Professeur(e),
Centre Armand Frappier Santé-Biotechnologie

Co-chercheuses et co-chercheurs

En provenance de l'INRS: Amadou Diogo Barry; Louise Henault-Ethier; Raphaelle Teysseire

En provenance de l'externe: Benoit Barbeau (École Polytechnique); Jonathan Chevrier (McGill) ; Pedro Segura (U de Sherbrooke); Jacques Brodeur (U de Montréal)

Financement(s) (# SOFE) : IRSC (UBR 304481)

Date d'approbation du projet : 03 avril 2024

Date d'entrée en vigueur du certificat : 03 avril 2024

Date d'échéance du certificat : 03 avril 2025

N. B. :

- Si votre projet se termine avant la date du prochain renouvellement, veuillez remplir le formulaire ***F9 - Fin de projet***.
- Si des modifications sont apportées à votre projet avant l'échéance du certificat, veuillez remplir le formulaire ***F8 - Modification de projet***.
- Tout nouveau membre de votre équipe de recherche devra être déclaré au CER-INRS lors de votre prochaine demande de renouvellement ou lors de la fin de votre projet si le renouvellement n'est pas requis.

Marie-Claude Proulx

Coordonatrice

Au nom de:

Xavier Leloup, Président

Comité d'éthique en recherche avec des êtres humain

Institut national de la recherche scientifique

Signé le 2024-04-03 à 16:14
