# Supplementary material for: A Multi-Pathway Approach to Pesticide Exposure in Agricultural Communities: Protocol for an Observational Study
Source: JMIR Res Protoc. 2026 Aug 21;15:e94740. doi: 10.2196/94740 (PMC13545533; doi:10.2196/94740)
Supplement: Supplementary file 5 [file resprot_v15i1e94740_app5.pdf]

**Review Type / Type d'évaluation:**	Reviewer 1/Évaluateur 1
**Name of Applicant / Nom du chercheur:**	Bouchard, Maryse
**Application No. / Numéro de demande:**	496494
**Agency / Agence:**	CIHR/IRSC
**Competition / Concours:**	Project Grant/Subvention Projet
**Committee / Comité:**	Public, Community & Population Health/Santé publique, santé communautaire et santé des populations
**Title / Titre:**	Exposition aux pesticides des communautés vivant à proximité de sites de production agricole intensive: Quantifier les sources d'exposition pour agir en prévention

Adjudication Criteria/Critères de sélection

Initial Score/Cote Initiale: 4.3

Top/Bottom Selection/Groupe supérieur/inférieur

- (●) **Top/Groupe supérieur**
- ( ) **Bottom/Groupe inférieur**

"This portion of your review has been translated by Canada's Translation Bureau according to your language preference. Should there be any discrepancies between this, and the original English version, the latter shall prevail."

« Cette partie de l'évaluation de votre proposition a été traduite par le Bureau de la traduction du Canada pour respecter vos préférences linguistiques. En cas de divergence entre les versions anglaise et française, la version anglaise originale prévaudra. »

**Review Type / Type d'évaluation:**	Reviewer 1/Évaluateur 1
**Name of Applicant / Nom du chercheur:**	Bouchard, Maryse
**Application No. / Numéro de demande:**	496494
**Agency / Agence:**	CIHR/IRSC
**Competition / Concours:**	Project Grant/Subvention Projet
**Committee / Comité:**	Public, Community & Population Health/Santé publique, santé communautaire et santé des populations
**Title / Titre:**	Exposition aux pesticides des communautés vivant à proximité de sites de production agricole intensive: Quantifier les sources d'exposition pour agir en prévention

Summary of Application/Résumé de la demande:
De nombreuses études ont montré la toxicité des pesticides, surtout pour les travailleurs agricoles fortement exposés. Or, jusqu'à 90 % des pesticides appliqués en champs se répandent dans l'environnement, soit par dérive, volatilisation ou ruissellement. Pourtant, ni au Québec ni ailleurs au Canada, il n'existe de données sur l'exposition des personnes vivant près des activités agricoles où sont épandus les pesticides. Chez un échantillon de personnes demeurant près de secteurs agricoles, les candidats proposent de quantifier le niveau de contamination de l'environnement intérieur et extérieur des résidences, de quantifier la dose d'exposition provenant de l'alimentation et de la consommation d'eau, de mesurer les résidus urinaires des pesticides des participants, et d'appliquer des méthodes analytiques de pointe pour mesurer plus de 100 pesticides différents dans ces échantillons.

Les résultats devraient permettre de déterminer l'importance respective des différentes sources d'exposition aux pesticides pour les personnes vivant en zone agricole, d'identifier les groupes de la communauté les plus exposés et les facteurs qui déterminent le niveau d'exposition.

**Review Type / Type d'évaluation:**	Reviewer 1/Évaluateur 1
**Name of Applicant / Nom du chercheur:**	Bouchard, Maryse
**Application No. / Numéro de demande:**	496494
**Agency / Agence:**	CIHR/IRSC
**Competition / Concours:**	Project Grant/Subvention Projet
**Committee / Comité:**	Public, Community & Population Health/Santé publique, santé communautaire et santé des populations
**Title / Titre:**	Exposition aux pesticides des communautés vivant à proximité de sites de production agricole intensive: Quantifier les sources d'exposition pour agir en prévention

Strengths and Weaknesses/Forces et faiblesses:

FORCES

Projet très pertinent;

Demande intéressante à lire et bien référencée;

Il sera possible de mesurer l'exposition à une centaine de molécules à la fois, ce qui était difficile et coûteux auparavant;

L'équipe de recherche est multidisciplinaire et comprends plusieurs types d'expertise. La candidate principale détient une chaire de recherche du Canada sur les contaminants environnementaux et la santé des populations;

La candidate principale est expérimentée en communication scientifique aux médias et au grand public;

Les candidats ont identifié une région spécifique du Québec où l'utilisation des pesticides est importante;

Les listes électorales seront utilisées pour identifier les ménages éligibles;

Le recrutement de tous les membres des ménages sélectionnés favorisera l'étude des variations d'exposition selon certaines caractéristiques, dont le sexe et le genre;

L'exposition sera quantifiée à partir d'échantillons biologiques recueillis sur le terrain par des membres de l'équipe de recherche;

L'utilisation de bracelets en silicone est originale et sera complémentaire aux autres moyens d'échantillonnage;

L'étude suivra les principes de la recherche participative basée sur la communauté (community-based participatory research) qui cherche à impliquer la communauté dans un échange d'informations qui mène à un apprentissage bidirectionnel entre les chercheurs et les citoyens. Un comité-aviseur sera impliqué dans toutes les phases de la recherche, incluant le raffinement des questions de recherche, le développement des protocoles et questionnaires, ainsi que l'interprétation et la communication des résultats. Le comité-aviseur sera formé de quatre membres de la communauté de différents âges et niveaux de littéracie;

Un retour aux participants est prévu, qui inclura les résultats globaux de l'étude et des résultats individuels : i) un rapport vulgarisé faisant état des principaux constats de l'étude et ii) un rapport présentant les résultats individuels des

concentrations de pesticides dans les échantillons. L'équipe organisera des rencontres d'information publiques ouvertes aux participants ainsi qu'aux membres de la communauté pour présenter les résultats de l'étude;

En cas de dépassement des seuils de toxicité, les participants concernés seront pris en charge par un médecin de la Clinique de médecine du travail et de l'environnement du Centre hospitalier de l'Université de Montréal;

Le projet comprend une stagiaire postdoctorale et deux étudiants à la maîtrise;

Le potentiel de l'étude pour identifier des facteurs de risque modifiables est important;

Le plan de transfert des connaissances est détaillé et identifie les principaux utilisateurs;

Je crois que les candidats ont très bien répondu à la plupart des commentaires des évaluateurs de la soumission précédente.

FAIBLESSES

L'utilisation des listes de téléphones filaires risque d'entraîner un biais de sélection;

Le questionnaire n'est pas encore validé.

**Review Type / Type d'évaluation:**	Reviewer 1/Évaluateur 1
**Name of Applicant / Nom du chercheur:**	Bouchard, Maryse
**Application No. / Numéro de demande:**	496494
**Agency / Agence:**	CIHR/IRSC
**Competition / Concours:**	Project Grant/Subvention Projet
**Committee / Comité:**	Public, Community & Population Health/Santé publique, santé communautaire et santé des populations
**Title / Titre:**	Exposition aux pesticides des communautés vivant à proximité de sites de production agricole intensive: Quantifier les sources d'exposition pour agir en prévention

Budget Recommendation/Recommandation budgétaire:

Le budget (910 000$: 182 000$ /an x 5 ans) me semble approprié. La candidate principale mentionne spécifiquement qu'il n'y a aucun recoupement financier de la présente demande avec ses autres projets financés.

**Review Type / Type d'évaluation:**	Reviewer 1/Évaluateur 1
**Name of Applicant / Nom du chercheur:**	Bouchard, Maryse
**Application No. / Numéro de demande:**	496494
**Agency / Agence:**	CIHR/IRSC
**Competition / Concours:**	Project Grant/Subvention Projet
**Committee / Comité:**	Public, Community & Population Health/Santé publique, santé communautaire et santé des populations
**Title / Titre:**	Exposition aux pesticides des communautés vivant à proximité de sites de production agricole intensive: Quantifier les sources d'exposition pour agir en prévention

Please indicate your appraisal of the integration of sex as a biological variable as a strength, weakness, or not applicable to the proposal./Prière de sélectionner une option pour donner votre évaluation de l'intégration du sexe comme variable biologique en tant que point fort ou point faible de la proposition, ou en tant qu'élément non applicable à la proposition.

- (●) **Strength/Point fort**
- ( ) **Weakness/Point faible**
- ( ) **Not applicable/Non applicable**

Please indicate your appraisal of the integration of gender as a socio-cultural determinant of health as a strength, weakness, or not applicable to the proposal./Prière de sélectionner une option pour donner votre évaluation de l'intégration du genre comme déterminant socioculturel de la santé en tant que point fort ou point faible de la proposition, ou en tant qu'élément non applicable à la proposition.

- (●) **Strength/Point fort**
- ( ) **Weakness/Point faible**
- ( ) **Not applicable/Non applicable**

Sex and/or Gender Considerations/Notions de sexe et/ou de genre:

Des mécanismes différents peuvent être impliqués dans l'exposition environnementale aux pesticides en fonction du sexe et du genre. Par exemple, plusieurs études sur l'exposition aux pesticides concernaient des inquiétudes pour la santé des enfants à naître, et portaient donc sur des femmes enceintes. Plusieurs travaux ont aussi été menées sur l'exposition des conjointes de travailleurs agricoles, car elles peuvent être exposées aux pesticides via les vêtements des hommes contaminés au travail. Ces deux exemples illustrent bien la différence entre le sexe (facteurs biologiques, physiologiques) et le genre, dont les candidats ont tenu compte adéquatement selon moi dans leur protocole.

**Review Type / Type d'évaluation:**	Reviewer 2/Évaluateur 2
**Name of Applicant / Nom du chercheur:**	Bouchard, Maryse
**Application No. / Numéro de demande:**	496494
**Agency / Agence:**	CIHR/IRSC
**Competition / Concours:**	Project Grant/Subvention Projet
**Committee / Comité:**	Public, Community & Population Health/Santé publique, santé communautaire et santé des populations
**Title / Titre:**	Exposition aux pesticides des communautés vivant à proximité de sites de production agricole intensive: Quantifier les sources d'exposition pour agir en prévention

Adjudication Criteria/Critères de sélection

Initial Score/Cote Initiale: 4.4

Top/Bottom Selection/Groupe supérieur/inférieur

- (●) **Top/Groupe supérieur**
- ( ) **Bottom/Groupe inférieur**

Review Type / Type d'évaluation: Reviewer 2/Évaluateur 2
Name of Applicant / Nom du chercheur: Bouchard, Maryse
Application No. / Numéro de demande: 496494
Agency / Agence: CIHR/IRSC
Competition / Concours: Project Grant/Subvention Projet
Committee / Comité: Public, Community & Population Health/Santé publique, santé communautaire et santé des populations
Title / Titre: Exposition aux pesticides des communautés vivant à proximité de sites de production agricole intensive: Quantifier les sources d'exposition pour agir en prévention

Summary of Application/Résumé de la demande:
Les pesticides sont très fréquemment utilisés en agriculture. Il est connu qu'une exposition élevée aux pesticides peut causer des problèmes de santé, mais le niveau d'exposition des humains aux pesticides demeure encore peu étudié. En particulier, il est vraisemblable que les personnes qui demeurent près des sites agricoles soient très exposées aux pesticides.

Ce projet de recherche vise à quantifier le niveau d'exposition aux pesticides de personnes vivant près des sites agricoles, mais qui ne sont pas en contact avec les pesticides dans le cadre de leur travail. L'exposition provenant de l'alimentation, de la consommation d'eau ainsi que de l'environnement intérieur et extérieur sera quantifiée.

Plus d'une centaine de pesticides seront mesurés par différents moyens. De nombreuses sources de données complémentaires seront collectées, dont des prélèvements d'urine, des échantillons des surfaces intérieures et extérieures, de l'eau du robinet, des bracelets en silicone (exposition respiratoire et dermale), ainsi que d'échantillonneurs passifs de l'eau du robinet pour un sous-échantillon. Des analyses descriptives permettront de décrire l'exposition des participants aux pesticides. Le niveau d'exposition sera comparé en fonction des caractéristiques des participants afin d'identifier les groupes les plus exposés à partir d'analyses de types ANOVA. Finalement, des régressions multivariables à effets mixtes serviront à estimer la contribution de chaque source d'exposition à l'exposition totale. Un rapport vulgarisé présentant les résultats globaux et individuels seront remis aux participants visant à la transparence tout en évitant de soulever des inquiétudes exagérées chez les participants. Les cas de dépassement des limites sécuritaires seront rapportés à la santé publique et les participants concernés seront référés à un médecin.

Il est attendu que ce projet comble un manque en connaissance majeur concernant l'exposition humaine aux pesticides et pourrait mener à des actions visant à protéger la santé des populations visées, selon les résultats obtenus.

Review Type / Type d'évaluation: Reviewer 2/Évaluateur 2
Name of Applicant / Nom du chercheur: Bouchard, Maryse
Application No. / Numéro de demande: 496494
Agency / Agence: CIHR/IRSC
Competition / Concours: Project Grant/Subvention Projet
Committee / Comité: Public, Community & Population Health/Santé publique, santé communautaire et santé des populations
Title / Titre: Exposition aux pesticides des communautés vivant à proximité de sites de production agricole intensive: Quantifier les sources d'exposition pour agir en prévention

---

Strengths and Weaknesses/Forces et faiblesses:

Forces :

-La nouvelle demande me semble avoir répondu de façon satisfaisante à la majorité des commentaires qui avaient été soulevés par les évaluateurs précédents.

-Le projet s'inscrit dans la continuité des travaux menés antérieurement par la personne candidate principale et son équipe.

-L'équipe dispose de l'expérience et l'expertise nécessaire pour réaliser le projet de recherche.

-Les partenariats nécessaires sont en place pour faciliter la réalisation du projet de recherche.

-La pertinence du projet est bien documentée. Les retombées du projet sont bien décrites. Il s'agit d'un projet avec fort potentiel d'impact sur les règlements et les politiques si ses résultats indiquent que les concentrations de polluant sont trop élevées.

-Le plan d'analyse tient compte de plusieurs défis qui pourraient être présents dans les données, dont les différentes sources de corrélation et la possible présence de données manquantes.

-Le plan de partage des connaissances a été révisé de façon à éviter de susciter de trop grandes inquiétudes chez les communautés visées si des concentrations de polluant trop élevées sont identifiées.

-Les considérations de sexe et de genre sont bien intégrées au plan de recherche.

Faiblesses :

-Le calcul de la taille d'échantillon demeure une faiblesse à mon avis. Bien que la nouvelle version présente un calcul beaucoup plus détaillé et tenant compte de la structure des données, les hypothèses sous-jacentes au calcul ne sont pas présentées. Plus précisément, comment les valeurs des paramètres utilisés dans le calcul de taille d'échantillon ont-ils été choisis ? Dans la demande, il est indiqué que 250 ménages permettront d'avoir une puissance suffisante pour détecter une différence « significative », mais toute différence peut être statistiquement significative avec suffisamment de données (et inversement, toute taille d'échantillon peut permettre de détecter une différence statistiquement significative si l'association est assez forte). Le calcul de taille d'échantillon serait plus convaincant s'il était basé sur des paramètres informés par la littérature et les connaissances scientifiques du domaine. Par exemple, le paramètre \beta_1 devrait correspondre à la taille d'association la plus petite qu'il serait pertinent d'être en mesure de détecter, mais le protocole indique que des valeurs de 0.3, 0.5 et 1.2 ont été utilisées selon les scénarios, sans expliquer le choix de ces valeurs. De façon similaire, la variance résiduelle a été fixée à 1 ou 2 selon les scénarios, mais sans justification de ces choix. Malheureusement, dans sa forme actuelle, le calcul de taille d'échantillon n'est pas du tout informatif à mon avis.

-Le nombre de ménages qui sera recruté n'est pas clairement indiqué. Il est inscrit que 250 ménages permettront d'atteindre une puissance suffisante pour les analyses globales, alors que 300 ménages seront nécessaires pour les analyses stratifiées, puis le budget prévoit une compensation pour 900 personnes. Doit-on déduire que 300 ménages seront recrutés et que l'on s'attend à avoir en moyenne 3 participants par ménage ?

**Review Type / Type d'évaluation:**	Reviewer 2/Évaluateur 2
**Name of Applicant / Nom du chercheur:**	Bouchard, Maryse
**Application No. / Numéro de demande:**	496494
**Agency / Agence:**	CIHR/IRSC
**Competition / Concours:**	Project Grant/Subvention Projet
**Committee / Comité:**	Public, Community & Population Health/Santé publique, santé communautaire et santé des populations
**Title / Titre:**	Exposition aux pesticides des communautés vivant à proximité de sites de production agricole intensive: Quantifier les sources d'exposition pour agir en prévention

Budget Recommendation/Recommandation budgétaire:

Le budget semble raisonnable.

**Review Type / Type d'évaluation:**	Reviewer 2/Évaluateur 2
**Name of Applicant / Nom du chercheur:**	Bouchard, Maryse
**Application No. / Numéro de demande:**	496494
**Agency / Agence:**	CIHR/IRSC
**Competition / Concours:**	Project Grant/Subvention Projet
**Committee / Comité:**	Public, Community & Population Health/Santé publique, santé communautaire et santé des populations
**Title / Titre:**	Exposition aux pesticides des communautés vivant à proximité de sites de production agricole intensive: Quantifier les sources d'exposition pour agir en prévention

Please indicate your appraisal of the integration of sex as a biological variable as a strength, weakness, or not applicable to the proposal./Prière de sélectionner une option pour donner votre évaluation de l'intégration du sexe comme variable biologique en tant que point fort ou point faible de la proposition, ou en tant qu'élément non applicable à la proposition.

- (●) **Strength/Point fort**
- ( ) **Weakness/Point faible**
- ( ) **Not applicable/Non applicable**

Please indicate your appraisal of the integration of gender as a socio-cultural determinant of health as a strength, weakness, or not applicable to the proposal./Prière de sélectionner une option pour donner votre évaluation de l'intégration du genre comme déterminant socioculturel de la santé en tant que point fort ou point faible de la proposition, ou en tant qu'élément non applicable à la proposition.

- (●) **Strength/Point fort**
- ( ) **Weakness/Point faible**
- ( ) **Not applicable/Non applicable**

**Review Type / Type d'évaluation:**	Reviewer 2/Évaluateur 2
**Name of Applicant / Nom du chercheur:**	Bouchard, Maryse
**Application No. / Numéro de demande:**	496494
**Agency / Agence:**	CIHR/IRSC
**Competition / Concours:**	Project Grant/Subvention Projet
**Committee / Comité:**	Public, Community & Population Health/Santé publique, santé communautaire et santé des populations
**Title / Titre:**	Exposition aux pesticides des communautés vivant à proximité de sites de production agricole intensive: Quantifier les sources d'exposition pour agir en prévention

Sex and/or Gender Considerations/Notions de sexe et/ou de genre:

Le sexe et le genre sont bien intégrés dans le projet de recherche. Des analyses stratifiées selon le sexe seront réalisées. Des analyses tenant compte de certains facteurs associés au genre sont aussi planifiées.

**Review Type / Type d'évaluation:**	Reviewer 3/Évaluateur 3
**Name of Applicant / Nom du chercheur:**	Bouchard, Maryse
**Application No. / Numéro de demande:**	496494
**Agency / Agence:**	CIHR/IRSC
**Competition / Concours:**	Project Grant/Subvention Projet
**Committee / Comité:**	Public, Community & Population Health/Santé publique, santé communautaire et santé des populations
**Title / Titre:**	Exposition aux pesticides des communautés vivant à proximité de sites de production agricole intensive: Quantifier les sources d'exposition pour agir en prévention

Adjudication Criteria/Critères de sélection

Initial Score/Cote Initiale: 4,3

Top/Bottom Selection/Groupe supérieur/inférieur

- (●) **Top/Groupe supérieur**
- ( ) **Bottom/Groupe inférieur**

Review Type / Type d'évaluation: Reviewer 3/Évaluateur 3
Name of Applicant / Nom du chercheur: Bouchard, Maryse
Application No. / Numéro de demande: 496494
Agency / Agence: CIHR/IRSC
Competition / Concours: Project Grant/Subvention Projet
Committee / Comité: Public, Community & Population Health/Santé publique, santé communautaire et santé des populations
Title / Titre: Exposition aux pesticides des communautés vivant à proximité de sites de production agricole intensive: Quantifier les sources d'exposition pour agir en prévention

Summary of Application/Résumé de la demande:

La présente demande consiste en fait en la nouvelle soumission d’un projet que j’ai eu le plaisir de lire l’an dernier. Le projet a déjà reçu une rétroaction très positive, mais des limites ont été cernées sur les plans méthodologique (échantillonnage, soutien communautaire), éthique (communication des niveaux élevés d’exposition) et statistique (taille de l’échantillon, techniques mieux adaptées aux données). Les auteurs ont répondu à tous les commentaires formulés et ont modifié plusieurs aspects du projet, notamment en ce qui concerne l’échantillonnage des participants et le lieu de l’étude. L’étude prévoit maintenant la création d’un comité de participation communautaire et l’intégration d’un biostatisticien dans l’équipe, et une vaste modification du plan a été réalisée relativement aux analyses statistiques.

La demande propose une étude novatrice et originale pour décrire et quantifier l’exposition aux pesticides agricoles des personnes vivant à proximité de terres agricoles traitées intensivement. Avec une utilisation totale citée de 115 millions de kg de pesticides par année (environ 3 kg par habitant), le Canada se situe quelque part au centre des comparaisons réalisées à l’échelle internationale quant à l’utilisation de pesticides.

Cette étude portera sur la gamme complète de pesticides qui pourraient avoir été utilisés sur ces terres.
L’exposition des participants à l’étude sera mesurée au niveau individuel, par des échantillons d’urine et un bracelet en silicone captant les pesticides dans l’air ambiant, au niveau résidentiel et environnemental, par des échantillons d’eau de puits, de sol et de poussière ambiante dans les résidences, et au niveau territorial, par géographie environnementale. Des analyses de laboratoire approfondies décriront ensuite l’exposition individuelle de chaque participant à un large éventail de catégories de pesticides. Enfin, la contribution respective des différentes sources d’exposition à la dose totale sera mesurée à l’aide des données urinaires. La dernière étape du projet vise à proposer des stratégies de prévention.

Review Type / Type d'évaluation: Reviewer 3/Évaluateur 3
Name of Applicant / Nom du chercheur: Bouchard, Maryse
Application No. / Numéro de demande: 496494
Agency / Agence: CIHR/IRSC
Competition / Concours: Project Grant/Subvention Projet
Committee / Comité: Public, Community & Population Health/Santé publique, santé communautaire et santé des populations
Title / Titre: Exposition aux pesticides des communautés vivant à proximité de sites de production agricole intensive: Quantifier les sources d'exposition pour agir en prévention

---

Strengths and Weaknesses/Forces et faiblesses:

Les forces et faiblesses vont comme suit :

- Longue tradition de collaboration complémentaire dans le cadre de projets de recherche semblables.
- Première étude visant à quantifier l'exposition à différents pesticides agricoles selon diverses sources d'exposition (excluant le travail agricole) dans des régions fortement agricoles et densément peuplées du Québec.
- Selon les résultats attendus, des moyens de réduire les expositions nocives seront proposés.

L'étude propose de constituer un échantillon aléatoire de tous les habitants de la localité choisie pour l'étude, selon les listes électorales, afin de réduire le biais de volontariat. Les enfants de plus de 4 ans des participants seront également invités à prendre part à l'étude. Des données sur l'exposition aux pesticides, y compris l'exposition alimentaire, ainsi que des données sur les conditions de vie et certaines données sur les antécédents médicaux seront recueillies. Peut-on s'attendre à ce que le type et la quantité des pesticides utilisés sur les lieux de l'étude varient en fonction des conditions météorologiques saisonnières et annuelles ou de la rotation des cultures? Si oui, comment cela sera-t-il pris en compte?

L'étude est principalement de nature descriptive et vise à combler un manque de connaissances sur l'exposition des personnes vivant à proximité de terres fortement traitées aux pesticides agricoles. La contribution des différentes sources d'exposition à l'ensemble de l'exposition sera analysée.
La participation représentative des habitants de la localité choisie, y compris les enfants vivant sur le lieu de l'étude, sera nécessaire pour assurer la validité des résultats. La participation d'un comité communautaire est une stratégie prometteuse.

**Review Type / Type d'évaluation:**	Reviewer 3/Évaluateur 3
**Name of Applicant / Nom du chercheur:**	Bouchard, Maryse
**Application No. / Numéro de demande:**	496494
**Agency / Agence:**	CIHR/IRSC
**Competition / Concours:**	Project Grant/Subvention Projet
**Committee / Comité:**	Public, Community & Population Health/Santé publique, santé communautaire et santé des populations
**Title / Titre:**	Exposition aux pesticides des communautés vivant à proximité de sites de production agricole intensive: Quantifier les sources d'exposition pour agir en prévention

Budget Recommendation/Recommandation budgétaire:

Le budget est en phase avec les activités de recherche proposées sur 4 ans.

**Review Type / Type d'évaluation:**	Reviewer 3/Évaluateur 3
**Name of Applicant / Nom du chercheur:**	Bouchard, Maryse
**Application No. / Numéro de demande:**	496494
**Agency / Agence:**	CIHR/IRSC
**Competition / Concours:**	Project Grant/Subvention Projet
**Committee / Comité:**	Public, Community & Population Health/Santé publique, santé communautaire et santé des populations
**Title / Titre:**	Exposition aux pesticides des communautés vivant à proximité de sites de production agricole intensive: Quantifier les sources d'exposition pour agir en prévention

Please indicate your appraisal of the integration of sex as a biological variable as a strength, weakness, or not applicable to the proposal./Prière de sélectionner une option pour donner votre évaluation de l'intégration du sexe comme variable biologique en tant que point fort ou point faible de la proposition, ou en tant qu'élément non applicable à la proposition.

- (●) **Strength/Point fort**
- ( ) **Weakness/Point faible**
- ( ) **Not applicable/Non applicable**

Please indicate your appraisal of the integration of gender as a socio-cultural determinant of health as a strength, weakness, or not applicable to the proposal./Prière de sélectionner une option pour donner votre évaluation de l'intégration du genre comme déterminant socioculturel de la santé en tant que point fort ou point faible de la proposition, ou en tant qu'élément non applicable à la proposition.

- (●) **Strength/Point fort**
- ( ) **Weakness/Point faible**
- ( ) **Not applicable/Non applicable**

**Review Type / Type d'évaluation:**	Reviewer 3/Évaluateur 3
**Name of Applicant / Nom du chercheur:**	Bouchard, Maryse
**Application No. / Numéro de demande:**	496494
**Agency / Agence:**	CIHR/IRSC
**Competition / Concours:**	Project Grant/Subvention Projet
**Committee / Comité:**	Public, Community & Population Health/Santé publique, santé communautaire et santé des populations
**Title / Titre:**	Exposition aux pesticides des communautés vivant à proximité de sites de production agricole intensive: Quantifier les sources d'exposition pour agir en prévention

Sex and/or Gender Considerations/Notions de sexe et/ou de genre:

L'équipe menant l'étude effectuera des analyses stratifiées selon le sexe et tiendra également compte des influences du genre sur l'exposition aux pesticides.

Review Type/Type d'évaluation: SO Notes /Notes de l'agent scientifique
Name of Applicant/Nom du chercheur: Bouchard, Maryse
Application No./Numéro de demande: 496494
Agency/Agence: CIHR/IRSC
Competition/Concours: 2023-03-08 Project Grant/Subvention Projet
Committee/Comité: Public, Community & Population Health/Santé publique, santé communautaire et santé des populations
Title/Titre: Exposition aux pesticides des communautés vivant à proximité de sites de production agricole intensive: Quantifier les sources d'exposition pour agir en prévention

Assessment/Évaluation:

Points forts (y compris les considérations relatives à l'ACSG)

- Comme on dispose de peu de données canadiennes sur l'exposition aux pesticides de la population des régions agricoles, il s'agit d'un projet très pertinent.
- Dans l'ensemble, la demande a très bien répondu aux examens précédents.
- La demande était bien rédigée et bien référencée.
- Solide équipe de recherche multidisciplinaire dirigée par la détentrice d'une chaire de recherche du Canada sur les contaminants environnementaux et la santé des populations.
- Les expositions seront caractérisées selon des facteurs liés au sexe et au genre.
- Le projet prévoit le recours à des approches novatrices d'échantillonnage biologique.
- L'approche fondée sur la science citoyenne a été bien décrite. Elle est novatrice et susceptible d'être efficace; dans l'ensemble, il y a une bonne participation des membres de la collectivité.
- Bonne intégration des stagiaires dans l'équipe d'étude.
- Le plan d'analyse a été bien décrit.
- Le plan d'AC est bien détaillé.

Points faibles (y compris les considérations relatives à l'ACSG)

- Une bonne utilisation des listes électorales pour le recrutement réduit le risque de biais de sélection, mais ce problème persiste même si l'étude n'est plus entièrement fondée sur la participation volontaire, comme c'était le cas auparavant.
- Des préoccupations ont été soulevées au sujet du calcul de la taille de l'échantillon. Le fondement des hypothèses relatives à la taille de l'échantillon n'est pas clair, ce qui limite l'utilité des calculs. Ces questions devraient être abordées.
- La définition de la taille de l'échantillon à recruter n'était pas claire (c.-à-d. le nombre de ménages, etc.). Les chiffres fournis dans la demande variaient, et une certaine incohérence générale a été observée.

**Review Type/Type d'évaluation:**	SO Notes /Notes de l'agent scientifique
**Name of Applicant/Nom du chercheur:**	Bouchard, Maryse
**Application No./Numéro de demande:**	496494
**Agency/Agence:**	CIHR/IRSC
**Competition/Concours:**	2023-03-08 Project Grant/Subvention Projet
**Committee/Comité:**	Public, Community & Population Health/Santé publique, santé communautaire et santé des populations
**Title/Titre:**	Exposition aux pesticides des communautés vivant à proximité de sites de production agricole intensive: Quantifier les sources d'exposition pour agir en prévention

Assessment/Évaluation:

Budget

- Aucun souci.

2023-06-02 01:32

“This portion of your review has been translated by Canada’s Translation Bureau according to your language preference. Should there be any discrepancies between this, and the original English version, the latter shall prevail.”
« Cette partie de l’évaluation de votre proposition a été traduite par le Bureau de la traduction du Canada pour respecter vos préférences linguistiques. En cas de divergence entre les versions anglaise et française, la version anglaise originale prévaudra. »
